# Supplementary figures and images for: Integrative multi-omics profiling reveals cAMP-independent mechanisms regulating hyphal morphogenesis in Candida albicans
Source: PLoS Pathog. 2021 Aug 16;17(8):e1009861. doi: 10.1371/journal.ppat.1009861 (PMC8389844; doi:10.1371/journal.ppat.1009861)

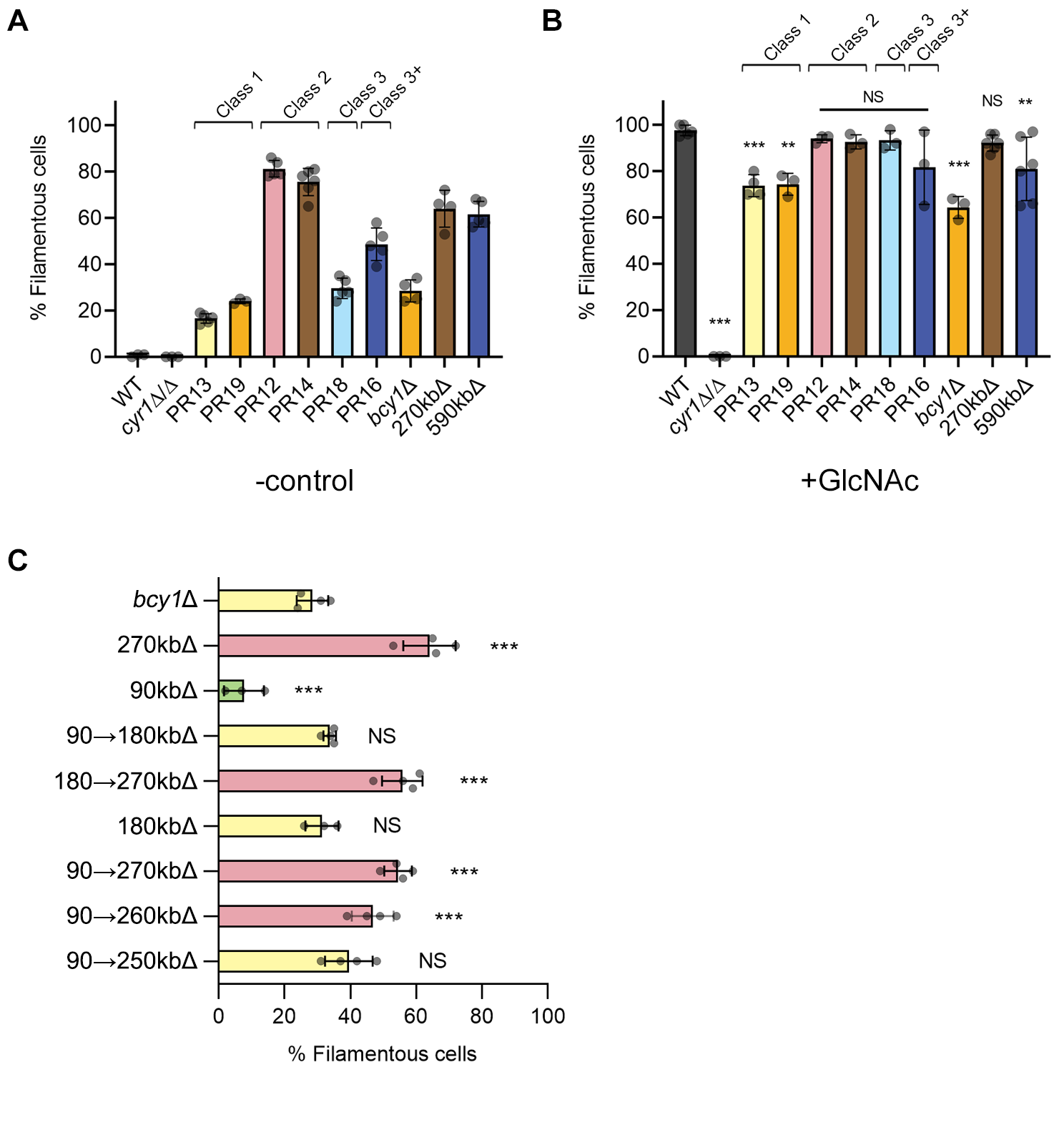

Supplement: S1 Fig — (A) The plot shows the percent of filamentous cells in YPD medium at 30°C (-control). (B) Graph indicating the percent of filamentous cells after growth in GlcNAc medium. Cells were grown in liquid medium containing 50 mM GlcNAc to induce hyphal growth at 37°C for 2 h and then filamentous cells were counted. (C) The percent of filamentous cells in YPD medium at 30°C; green, weak filamentation; yellow, intermediate filamentation; pink, strong filamentation. Deletions indicated on the left are on chromosome 2 and they are heterozygous; the cells retain a wild-type version of chromosome 2. (A, B, and C) Shown is the mean ± SD of at least 3 independent experiments with at least 100 cells counted for each condition. Statistical analysis was performed using one-way ANOVA with Dunnett’s multiple comparisons test comparing the strains with the WT or parental strain; NS p > 0.05, ** p < 0.01, *** p < 0.001. (TIF) [file ppat.1009861.s001.tif]

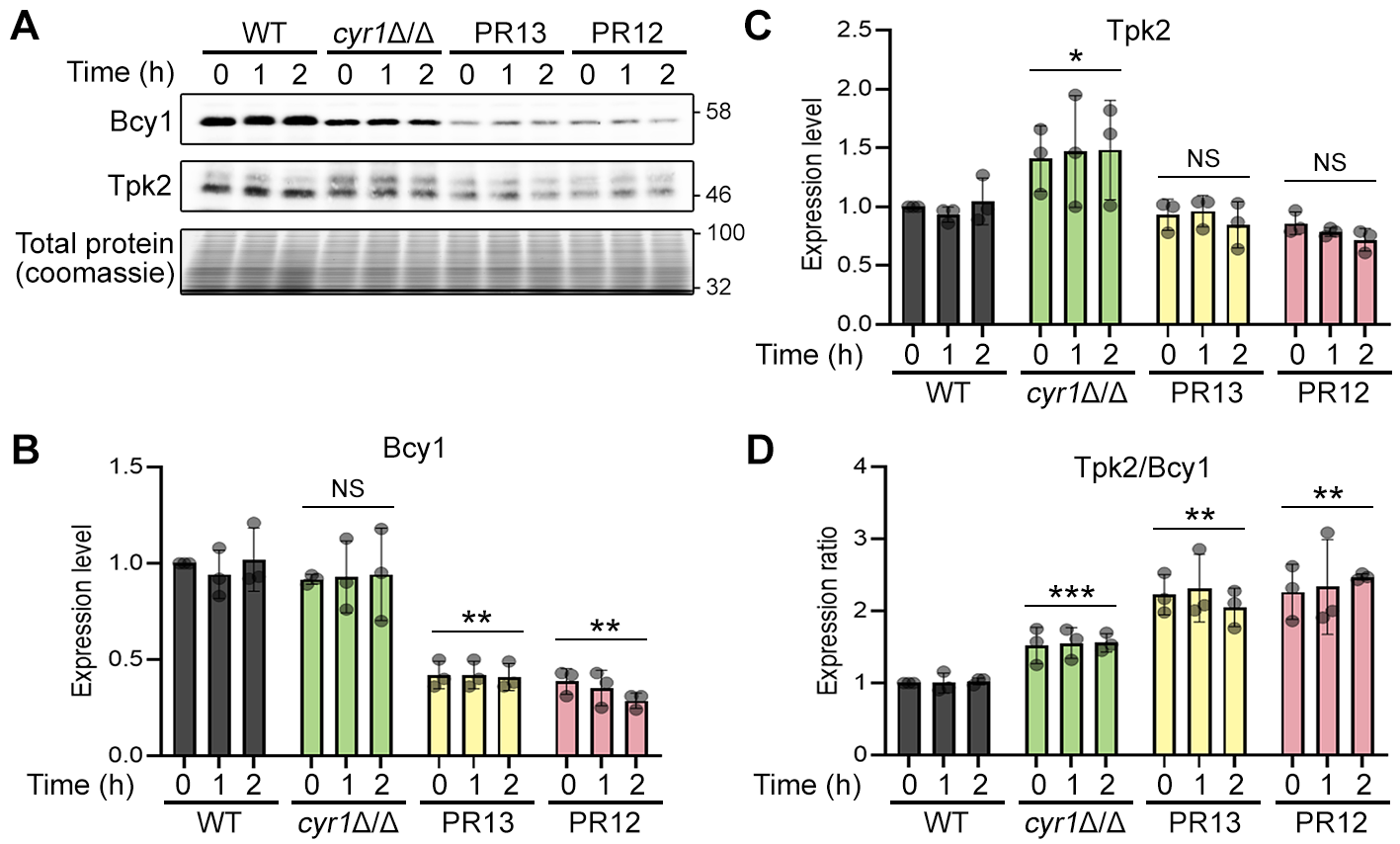

Supplement: S2 Fig — (A) Western blot detection of the negative regulatory subunit (Bcy1) and the catalytic subunit (Tpk2) of PKA. Cells were grown at 37°C in liquid galactose medium and then 50 mM GlcNAc was added for 2 h to induce hyphae. The sizes of the protein standards (kDa) are indicated on the right of each blot. Images shown are representative of three independent experiments. (B-D) Relative levels of Bcy1 (B), Tpk2 (C), and Tpk2/Bcy1 ratio (D) compared to the WT 0-h samples. Shown is the mean ± SD of 3 independent experiments. Expression levels were normalized to total proteins on Coomassie-stained gels. Statistical analysis was performed using one-way ANOVA with Dunnett’s multiple comparisons test comparing the strains with the WT; NS p > 0.05, * p < 0.05, ** p < 0.01, *** p < 0.001. (TIF) [file ppat.1009861.s002.tif]

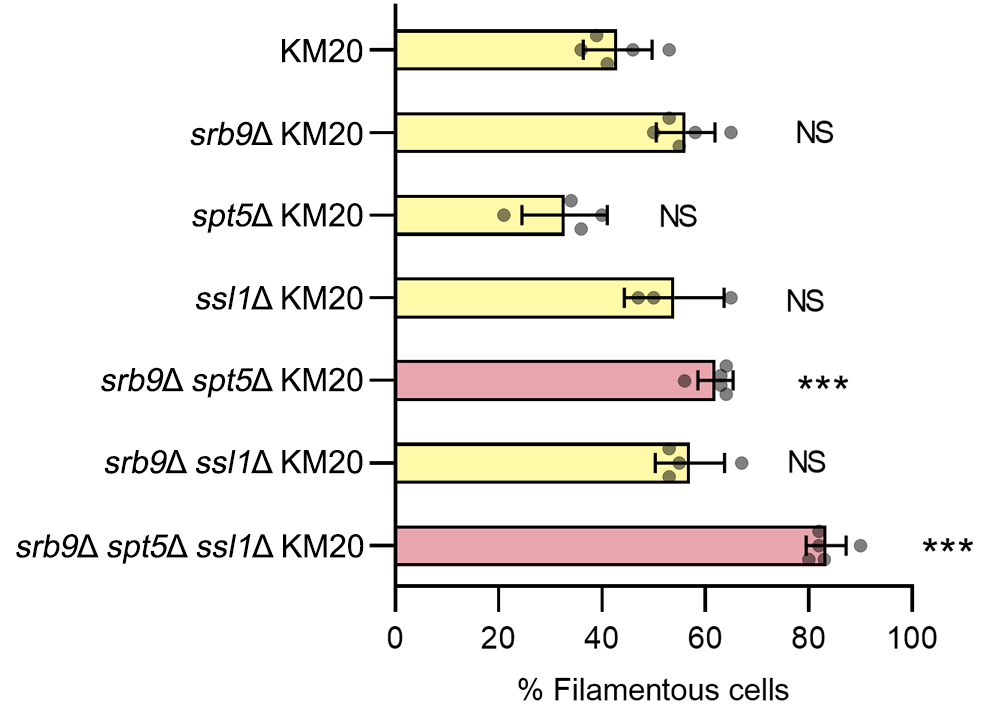

Supplement: S3 Fig — The single, double, and triple heterozygous deletion mutants of SRB9, SPT5, and SSL1 were created in KM20 strain background (Chr2L 90kb→250kbΔ bcy1Δ/BCY1 cyr1Δ/Δ). The plot shows the percent of filamentous cells in GlcNAc medium. Cells were grown in liquid medium containing 50 mM GlcNAc to induce hyphal growth at 37°C for 2 h and then filamentous cells were counted.; yellow, intermediate hyphal induction; pink, strong hyphal induction. Statistical analysis was performed using one-way ANOVA with Dunnett’s multiple comparisons test comparing the strains with the parental strain; NS p > 0.01, *** p < 0.001. (TIF) [file ppat.1009861.s003.tif]

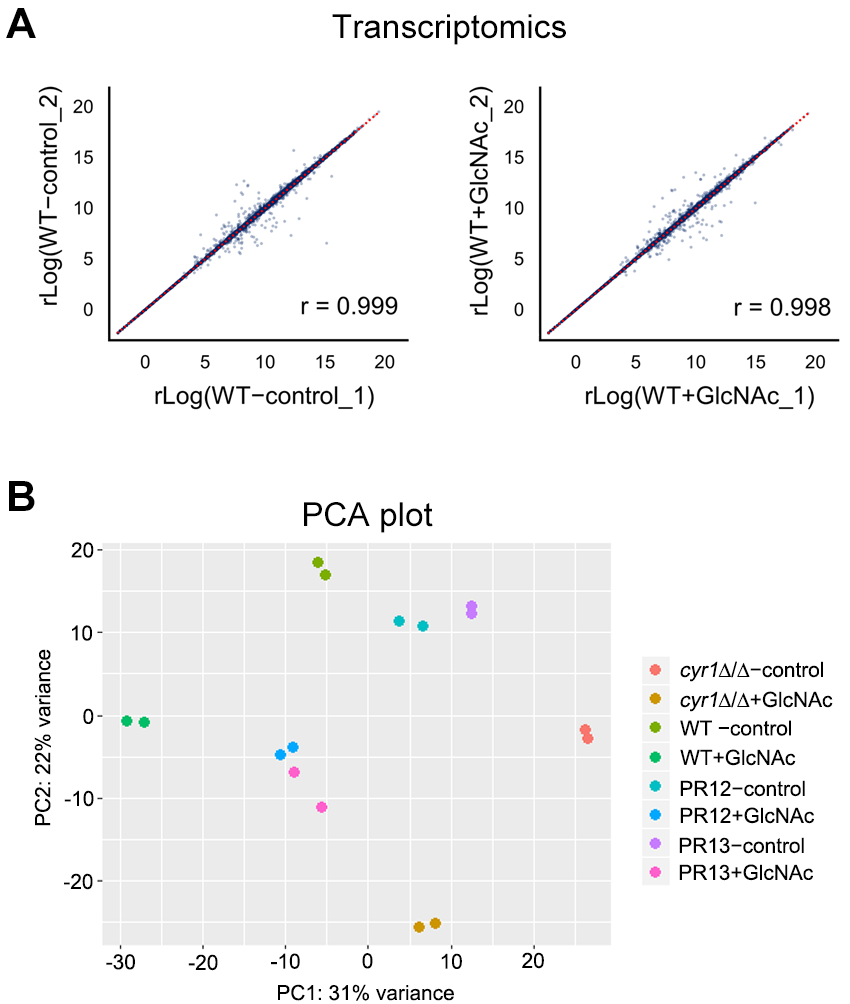

Supplement: S4 Fig — (A) Representative null comparisons of the biological replicates show very high reproducibility (r ≈ 1.00) in the RNA-seq dataset. Each dot represents the transcript level of individual gene in the scatter plots. We compared the biological replicates of WT−control and WT+GlcNAc. rLog, regularized log transformation. (B) PCA plot shows clusters of biological replicates based on their similarity in transcriptome. (TIF) [file ppat.1009861.s004.tif]

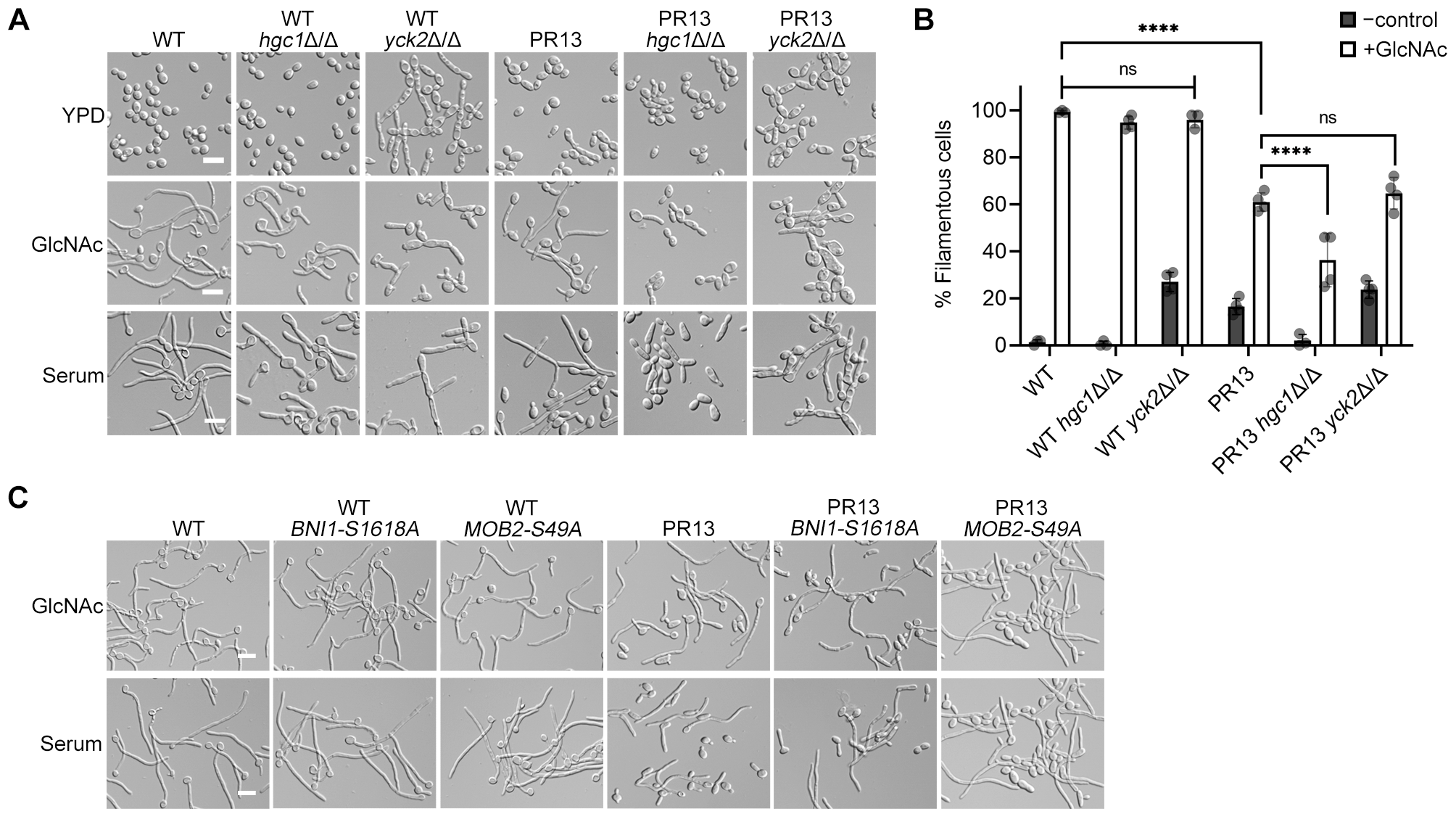

Supplement: S5 Fig — (A) Deletion of Cdc28 cyclin (HGC1) and casein kinase 1 (YCK2) disrupt normal hyphal growth in WT and PR13 backgrounds. (B) The plot shows the percent of filamentous cells in YPD medium at 30°C (-control) and after 2-h growth in GlcNAc medium at 37°C (+GlcNAc). Shown is the mean ± SD of at least 3 independent experiments with at least 100 cells counted for each condition. Statistical analysis was performed using one-way ANOVA with Dunnett’s multiple comparisons test comparing the strains with the WT or parental strain; ns p > 0.05, **** p < 0.0001. (C) Phospho-mutants of Mob2 and Bni1 did not show an obvious defect in hyphal growth. (A and C) The strains indicated at the top were grown in the liquid medium indicated on the left, and then hyphal induction was assessed microscopically. Cells were grown in liquid medium containing 15% serum or 50 mM N-acetylglucosamine (GlcNAc) to induce hyphal growth. Cells were incubated at 37°C for 2 h and then photographed. Scale bar, 10 μm. (TIF) [file ppat.1009861.s005.tif]

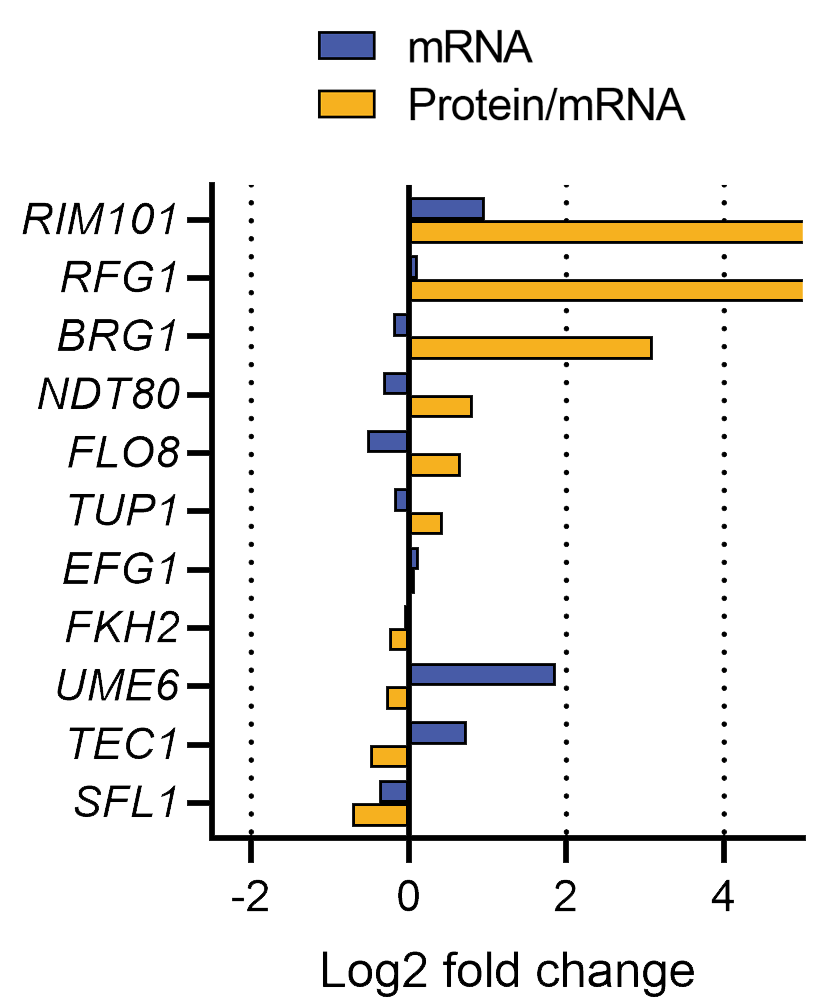

Supplement: S6 Fig — The relative change in protein-to-mRNA ratio for the selected 11 hyphal regulators TFs is shown in yellow, relative changes in mRNA expression are shown in blue. The protein-to-mRNA ratio of RIM101 and RFG1 increased dramatically (log2 fold change > 4) during hyphal induction while mRNA levels did not. (TIF) [file ppat.1009861.s006.tif]
