# Supplementary material for: Integrative multi-omics profiling reveals cAMP-independent mechanisms regulating hyphal morphogenesis in Candida albicans
Source: PLoS Pathog. 2021 Aug 16;17(8):e1009861. doi: 10.1371/journal.ppat.1009861 (PMC8389844; doi:10.1371/journal.ppat.1009861)
Supplement: S1 Table — a 23-bp telomere repeat sequence, CACCAAGAAGTTAGACATCCGTA. (DOCX) [file ppat.1009861.s007.docx]

**S1 Table. Genome analysis summary**

| Strain | PR Class |  | Short genotype | *de novo* telomere addition^a^ | Telomere seed sequence |
| --- | --- | --- | --- | --- | --- |
| *cyr1*∆/∆ | - |  | *cyr1*∆/∆ | - | - |
| PR13 | 1 |  | *bcy1-Q82**/*BCY1 cyr1∆*/*∆* | - | - |
| PR19 | 1 |  | *bcy1-E19**/*BCY1 cyr1∆*/*∆* | - | - |
| PR12 | 2 |  | *Monosomy of ~276 kb of Chr2L cyr1∆*/*∆* | Detected at Chr2: 276,278 | ACATCCGTA |
| PR14 | 2 |  | *Monosomy of ~276 kb of Chr2L cyr1∆*/*∆* | Detected at Chr2: 276,278 | ACATCCGTA |
| PR18 | 3 |  | *Monosomy of ~590 kb of Chr2L cyr1∆*/*∆* | Detected at Chr2: 590,175 | CATCCG |
| PR16 | 3+ |  | *Monosomy of ~557 kb of Chr2L*  *Trisomy of ~1342 kb of Chr2 cyr1∆*/*∆* | Detected at Chr2: 1,899,373 | GAAG |
| PR2 | 3+ |  | *Monosomy of ~557 kb of Chr2L*  *Trisomy of ~1239 kb of Chr2 cyr1∆*/*∆* | Detected at Chr2: 1,796,423 | CGTACA |

^a^ 23-bp telomere repeat sequence, CACCAAGAAGTTAGACATCCGTA
