# Supplementary material for: Integrative multi-omics profiling reveals cAMP-independent mechanisms regulating hyphal morphogenesis in Candida albicans
Source: PLoS Pathog. 2021 Aug 16;17(8):e1009861. doi: 10.1371/journal.ppat.1009861 (PMC8389844; doi:10.1371/journal.ppat.1009861)
Supplement: S2 Table — a Descriptions and deletion phenotypes were obtained from Candida Genome Database. (DOCX) [file ppat.1009861.s008.docx]

**S2 Table. Potential phosphorylation substrates of Cdc28 and Yck2 during hyphal induction**

| Gene | Phosphosite | Description^a^ | Deletion phenotype^a^ |
| --- | --- | --- | --- |
| *BNI4* | S523 | Formin; Protein required for wild-type cell wall chitin distribution, morphology, hyphal growth | Hyphal induction: normal |
| *FPK1* | T143, S337 | Serine/threonine protein kinase | Hyphal induction: normal |
| *ZDS1* | S272 | Protein with a role in regulating Swe1p-dependent polarized growth | Hyphal induction: normal |
| *INT1* | S416, T1214 | Bud site selection protein Bud4 | Hyphal induction: normal |
| *SOL1* | T16 | Cell cycle regulator | Hyphal induction: normal |
| *BNI1* | S1619 | Formin; Role in cytoskeletal organization, cell polarity | Hyphal induction: abnormal |
| *MOB2* | S49 | Cbk1 kinase activator protein | Hyphal induction: absent |
| *ASK1* | S249 | Essential subunit of the Dam1 (DASH) complex, which acts in chromosome segregation by coupling kinetochores to spindle microtubules | Inviable |

^a^ Descriptions and deletion phenotypes were obtained from *Candida* Genome Database.
