# Supplementary material for: Integrative multi-omics profiling reveals cAMP-independent mechanisms regulating hyphal morphogenesis in Candida albicans
Source: PLoS Pathog. 2021 Aug 16;17(8):e1009861. doi: 10.1371/journal.ppat.1009861 (PMC8389844; doi:10.1371/journal.ppat.1009861)
Supplement: S3 Table — (DOCX) [file ppat.1009861.s009.docx]

**S3 Table. *C. albicans* strains used in this study**

| **Strain** | **Short genotype** | **Parent or reference** | **Genotype** |
| --- | --- | --- | --- |
| DIC185 | Prototrophic wild type strain | (Wilson et al., 1999) | *ura3∆::λimm434*/*URA3 his1::hisG*/*HIS1 arg4::hisG*/*ARG4* |
| SP60-66 | *cyr1∆*/*∆* | (Parrino et al., 2017) | *cyr1∆::FRT*/*cyr1∆::ARG4 ura3∆::λimm434*/*URA3 his1::hisG*/*HIS1 arg4::hisG*/*arg4::hisG* |
| PR2 | *Monosomy of ~557 kb of Chr2L*  *Trisomy of ~1239 kb of Chr2*  *cyr1∆*/*∆* | SP60-66 | *Monosomy of ~557 kb of Chr2L (Chr2L→C2_02790C)*  *Trisomy of ~1239 kb of Chr2 (C2_02800W→C2_08860W)*  *cyr1∆::FRT*/*cyr1∆::ARG4 ura3∆::λimm434*/*URA3 his1::hisG*/*HIS1 arg4::hisG*/*arg4::hisG* |
| PR12 | *Monosomy of ~276 kb of Chr2L*  *cyr1∆*/*∆* | SP60-66 | *Monosomy of ~276 kb of Chr2L (Chr2L→C2_01540W)*  *cyr1∆::FRT*/*cyr1∆::ARG4 ura3∆::λimm434*/*URA3 his1::hisG*/*HIS1 arg4::hisG*/*arg4::hisG* |
| PR13 | *bcy1-Q82**/*BCY1 cyr1∆*/*∆* | SP60-66 | *bcy1-Q82**/*BCY1 cyr1∆::FRT*/*cyr1∆::ARG4 ura3∆::λimm434*/*URA3 his1::hisG*/*HIS1 arg4::hisG*/*arg4::hisG* |
| PR14 | *Monosomy of ~276 kb of Chr2L*  *cyr1∆*/*∆* | SP60-66 | *Monosomy of ~276 kb of Chr2L (Chr2L→C2_01540W)*  *cyr1∆::FRT*/*cyr1∆::ARG4 ura3∆::λimm434*/*URA3 his1::hisG*/*HIS1 arg4::hisG*/*arg4::hisG* |
| PR16 | *Monosomy of ~557 kb of Chr2L*  *Trisomy of ~1342 kb of Chr2*  *cyr1∆*/*∆* | SP60-66 | *Monosomy of ~557 kb of Chr2L (Chr2L→C2_02790C)*  *Trisomy of ~1342 kb of Chr2 (C2_02800W→C2_09290W)*  *cyr1∆::FRT*/*cyr1∆::ARG4 ura3∆::λimm434*/*URA3 his1::hisG*/*HIS1 arg4::hisG*/*arg4::hisG* |
| PR18 | *Monosomy of ~590 kb of Chr2L*  *cyr1∆*/*∆* | SP60-66 | *Monosomy of ~590 kb of Chr2L (Chr2L→C2_02960C)*  *cyr1∆::FRT*/*cyr1∆::ARG4 ura3∆::λimm434*/*URA3 his1::hisG*/*HIS1 arg4::hisG*/*arg4::hisG* |
| PR19 | *bcy1-E19**/*BCY1 cyr1∆*/*∆* | SP60-66 | *bcy1-E19**/*BCY1 cyr1∆::FRT*/*cyr1∆::ARG4 ura3∆::λimm434*/*URA3 his1::hisG*/*HIS1 arg4::hisG*/*arg4::hisG* |
| KM11 | *bcy1∆*/*BCY1 cyr1∆*/*∆* | SP60-66 | *bcy1∆::SAT1-FLIP*/*BCY1 cyr1∆::FRT*/*cyr1∆::ARG4 ura3∆::λimm434*/*URA3 his1::hisG*/*HIS1 arg4::hisG*/*arg4::hisG* |
| KM12 | *Monosomic:C2_00030W→C2_01540W*  *(Chr2L 270kb deletion)*  *bcy1∆*/*BCY1 cyr1∆*/*∆* | SP60-66 | *c2_00030w→c2_01540wΔ::SAT1-FLIP*/*C2_00030W→C2_01540W cyr1∆::FRT*/*cyr1∆::ARG4 ura3∆::λimm434*/*URA3 his1::hisG*/*HIS1 arg4::hisG*/*arg4::hisG* |
| KM13 | *Monosomic:C2_00030W→C2_02960C*  *(Chr2L 590kb deletion)*  *bcy1∆*/*BCY1 cyr1∆*/*∆* | SP60-66 | *c2_00030w→c2_02960cΔ::SAT1-FLIP*/*C2_00030W→C2_02960C cyr1∆::FRT*/*cyr1∆::ARG4 ura3∆::λimm434*/*URA3 his1::hisG*/*HIS1 arg4::hisG*/*arg4::hisG* |
| KM14 | *Monosomic:C2_00030W→C2_00550W*  *(Chr2L 90kb deletion)*  *bcy1∆*/*BCY1 cyr1∆*/*∆* | KM11 | *c2_00030w→c2_00550wΔ::SAT1-FLIP*/*C2_00030W→C2_00550W cyr1∆::FRT*/*cyr1∆::ARG4 ura3∆::λimm434*/*URA3 his1::hisG*/*HIS1 arg4::hisG*/*arg4::hisG* |
| KM15 | *Monosomic:C2_00560W→C2_01140C*  *(Chr2L 90kb→180kb deletion)*  *bcy1∆*/*BCY1 cyr1∆*/*∆* | KM11 | *c2_00560w→c2_01140cΔ::SAT1-FLIP*/*C2_00560W→C2_01140C cyr1∆::FRT*/*cyr1∆::ARG4 ura3∆::λimm434*/*URA3 his1::hisG*/*HIS1 arg4::hisG*/*arg4::hisG* |
| KM16 | *Monosomic:C2_01150W→C2_01540W*  *(Chr2L 180kb→270kb deletion)*  *bcy1∆*/*BCY1 cyr1∆*/*∆* | KM11 | *c2_01150w→c2_01540wΔ::SAT1-FLIP*/*C2_01150W→C2_01540W cyr1∆::FRT*/*cyr1∆::ARG4 ura3∆::λimm434*/*URA3 his1::hisG*/*HIS1 arg4::hisG*/*arg4::hisG* |
| KM17 | *Monosomic:C2_00030W→C2_01140C*  *(Chr2L 180kb deletion)*  *bcy1∆*/*BCY1 cyr1∆*/*∆* | KM11 | *c2_00030w→c2_01140cΔ::SAT1-FLIP*/*C2_00030W→C2_01140C cyr1∆::FRT*/*cyr1∆::ARG4 ura3∆::λimm434*/*URA3 his1::hisG*/*HIS1 arg4::hisG*/*arg4::hisG* |
| KM18 | *Monosomic:C2_00560W→C2_01540W*  *(Chr2L 90kb→270kb deletion)*  *bcy1∆*/*BCY1 cyr1∆*/*∆* | KM11 | *c2_00560w→c2_01540wΔ::SAT1-FLIP*/*C2_00560W→C2_01540W cyr1∆::FRT*/*cyr1∆::ARG4 ura3∆::λimm434*/*URA3 his1::hisG*/*HIS1 arg4::hisG*/*arg4::hisG* |
| KM19 | *Monosomic:C2_00560W→C2_01500W*  *(Chr2L 90kb→260kb deletion)*  *bcy1∆*/*BCY1 cyr1∆*/*∆* | KM11 | *c2_00560w→c2_01500wΔ::SAT1-FLIP*/*C2_00560W→C2_01500W cyr1∆::FRT*/*cyr1∆::ARG4 ura3∆::λimm434*/*URA3 his1::hisG*/*HIS1 arg4::hisG*/*arg4::hisG* |
| KM20 | *Monosomic:C2_00560W→C2_01460C*  *(Chr2L 90kb→250kb deletion)*  *bcy1∆*/*BCY1 cyr1∆*/*∆* | KM11 | *c2_00560w→c2_01460cΔ::SAT1-FLIP*/*C2_00560W→C2_01460C cyr1∆::FRT*/*cyr1∆::ARG4 ura3∆::λimm434*/*URA3 his1::hisG*/*HIS1 arg4::hisG*/*arg4::hisG* |
| KM21 | *hgc1∆*/*∆* | DIC185 | *hgc1∆::SAT1-FLIP*/*hgc1∆::SAT1-FLIP ura3∆::λimm434*/*URA3 his1::hisG*/*HIS1 arg4::hisG*/*ARG4* |
| KM22 | *yck2∆*/*∆* | DIC185 | *yck2∆::SAT1-FLIP*/*yck2∆::SAT1-FLIP ura3∆::λimm434*/*URA3 his1::hisG*/*HIS1 arg4::hisG*/*ARG4* |
| KM23 | *hgc1∆*/*∆* PR13 | PR13 | *hgc1∆::SAT1-FLIP*/*hgc1∆::SAT1-FLIP bcy1-Q82**/*BCY1 cyr1∆::FRT*/*cyr1∆::ARG4 ura3∆::λimm434*/*URA3 his1::hisG*/*HIS1 arg4::hisG*/*arg4::hisG* |
| KM24 | *yck2∆*/*∆* PR13 | PR13 | *yck2∆::SAT1-FLIP*/*yck2∆::SAT1-FLIP bcy1-Q82**/*BCY1 cyr1∆::FRT*/*cyr1∆::ARG4 ura3∆::λimm434*/*URA3 his1::hisG*/*HIS1 arg4::hisG*/*arg4::hisG* |
| KM25 | *BNI1-S1618A* | DIC185 | *BNI1-S1618A*/*BNI1-S1618A eno1::CaCAS9-SAT1-sgRNA*/*ENO1 ura3∆::λimm434*/*URA3 his1::hisG*/*HIS1 arg4::hisG*/*ARG4* |
| KM26 | *MOB2-S49A* | DIC185 | *MOB2-S49A*/*MOB2-S49A eno1::CaCAS9-SAT1-sgRNA*/*ENO1 ura3∆::λimm434*/*URA3 his1::hisG*/*HIS1 arg4::hisG*/*ARG4* |
| KM27 | *BNI1-S1618A* PR13 | PR13 | *BNI1-S1618A*/*BNI1-S1618A eno1::CaCAS9-SAT1-sgRNA*/*ENO1 bcy1-Q82**/*BCY1 cyr1∆::FRT*/*cyr1∆::ARG4 ura3∆::λimm434*/*URA3 his1::hisG*/*HIS1 arg4::hisG*/*arg4::hisG* |
| KM28 | *MOB2-S49A* PR13 | PR13 | *MOB2-S49A*/*MOB2-S49A eno1::CaCAS9-SAT1-sgRNA*/*ENO1 bcy1-Q82**/*BCY1 cyr1∆::FRT*/*cyr1∆::ARG4 ura3∆::λimm434*/*URA3 his1::hisG*/*HIS1 arg4::hisG*/*arg4::hisG* |
| KM29 | *srb9∆* KM20 | KM20 | *srb9∆::SAT1-FLIP*/*SRB9* c2*_00560w→c2_01460cΔ::SAT1-FLIP*/*C2_00560W→C2_01460C*  *cyr1∆::FRT*/*cyr1∆::ARG4 ura3∆::λimm434*/*URA3 his1::hisG*/*HIS1 arg4::hisG*/*arg4::hisG* |
| KM30 | *spt5∆* KM20 | KM20 | *spt5∆::SAT1-FLIP*/*SPT5 c2_00560w→c2_01460cΔ::SAT1-FLIP*/*C2_00560W→C2_01460C*  *cyr1∆::FRT*/*cyr1∆::ARG4 ura3∆::λimm434*/*URA3 his1::hisG*/*HIS1 arg4::hisG*/*arg4::hisG* |
| KM31 | *ssl1∆* KM20 | KM20 | *ssl1∆::SAT1-FLIP*/*SSL1 c2_00560w→c2_01460cΔ::SAT1-FLIP*/*C2_00560W→C2_01460C*  *cyr1∆::FRT*/*cyr1∆::ARG4 ura3∆::λimm434*/*URA3 his1::hisG*/*HIS1 arg4::hisG*/*arg4::hisG* |
| KM32 | *srb9∆* *spt5∆* KM20 | KM20 | *srb9∆::SAT1-FLIP*/*SRB9* *spt5∆::SAT1-FLIP*/*SPT5 c2_00560w→c2_01460cΔ::SAT1-FLIP*/*C2_00560W→C2_01460C cyr1∆::FRT*/*cyr1∆::ARG4 ura3∆::λimm434*/*URA3 his1::hisG*/*HIS1 arg4::hisG*/*arg4::hisG* |
| KM33 | *srb9∆* *ssl1∆* KM20 | KM20 | *srb9∆::SAT1-FLIP*/*SRB9* *ssl1∆::SAT1-FLIP*/*SSL1 c2_00560w→c2_01460cΔ::SAT1-FLIP*/*C2_00560W→C2_01460C cyr1∆::FRT*/*cyr1∆::ARG4 ura3∆::λimm434*/*URA3 his1::hisG*/*HIS1 arg4::hisG*/*arg4::hisG* |
