# Supplementary material for: Integrative multi-omics profiling reveals cAMP-independent mechanisms regulating hyphal morphogenesis in Candida albicans
Source: PLoS Pathog. 2021 Aug 16;17(8):e1009861. doi: 10.1371/journal.ppat.1009861 (PMC8389844; doi:10.1371/journal.ppat.1009861)
Supplement: S4 Table — (DOCX) [file ppat.1009861.s010.docx]

**S4 Table. Target sequences of the sgRNAs**

| **Target gene** | **20-nt target sequence** |
| --- | --- |
| *C2_00030W* | TCAAGACGATCTGAAATTGG |
| *C2_01540W* | GAGCCAAAAGATACAAGCGG |
| *C2_02960C* | TTGTAGCCAAGTCATACCCG |
| *C2_00550W* | TCATCAAAAAGAGCCAGTGT |
| *C2_00560W* | GATGCTATCGAGGAAGAAGT |
| *C2_01140C* | TTCAACATAGAAGTCCATAT |
| *C2_01150W* | TTTAATTGTAACAAGTACCG |
| *C2_01300C* | ATGATGATAAAGTTGACCGT |
| *C2_01310W* | ATGGGTGGGATTATTAATGT |
| *C2_01430W* | AAAGCTTTCAAATTACACGT |
| *C2_01420C* | AACAGATATAGTCTATGCTA |
| *C2_01460C* | GTTAAATATTAAAAAGTGCT |
| *C2_01500W* | TGTATTTCAATATATGCCAA |
| *C2_00770W* | AGATAAAGCTTTAGACATTG |
| *C2_01000W* | CTCATCAACATCTACAGCTG |
| *HGC1* | GTAGTACTACATGATGAACT |
| *YCK2* | CCAGCAGTTACATTATGTGA |
