# Supplementary material for: Integrative multi-omics profiling reveals cAMP-independent mechanisms regulating hyphal morphogenesis in Candida albicans
Source: PLoS Pathog. 2021 Aug 16;17(8):e1009861. doi: 10.1371/journal.ppat.1009861 (PMC8389844; doi:10.1371/journal.ppat.1009861)
Supplement: S5 Table — (DOCX) [file ppat.1009861.s011.docx]

**S5 Table. The oligonucleotides used in this study**

| **Name** | **Sequence (5' to 3')** | **Description** |
| --- | --- | --- |
| SNR52_R_30 | ccaatttcagatcgtcttgaCAAATTAAAAATAGTTTACGCAAGTC | sgRNA synthesis against *C2_00030W* |
| sgRNA_F_30 | tcaagacgatctgaaattggGTTTTAGAGCTAGAAATAGCAAGTTAAA | sgRNA synthesis against *C2_00030W* |
| SNR52_R_1540 | ccgcttgtatcttttggctcCAAATTAAAAATAGTTTACGCAAGTC | sgRNA synthesis against *C2_01540W* |
| sgRNA_F_1540 | gagccaaaagatacaagcggGTTTTAGAGCTAGAAATAGCAAGTTAAA | sgRNA synthesis against *C2_01540W* |
| SNR52_R_2960 | cgggtatgacttggctacaaCAAATTAAAAATAGTTTACGCAAGTC | sgRNA synthesis against *C2_02960C* |
| sgRNA_F_2960 | ttgtagccaagtcatacccgGTTTTAGAGCTAGAAATAGCAAGTTAAA | sgRNA synthesis against *C2_02960C* |
| 30_NAT_FLP_For | ctagaaaccgatgccttgcccatcaacaccatcacagataaaatcaatctccccattggccaccccgaatcaatcacactTAAAGGGAACAAAAGCTGGG | Deletion construct with a homology arm to *C2_00030W* |
| 1540_NAT_FLP_Rev | cttgtggttgagatttcacagagcaaccatctttcttcaaaacagactgatcatgtctaaactctttaccatgaccataaCTCTAGAACTAGTGGATCTG | Deletion construct with a homology arm to *C2_01540W* |
| 2960_NAT_FLP_Rev | tggatctctagaacattttgaaaatacagcaatagacccggataccatttttcaggagaagggtgtaattgaattgaacaCTCTAGAACTAGTGGATCTG | Deletion construct with a homology arm to *C2_02960C* |
| Chr2Del_F1 | TACCGGTCTTCCCATCAACT | Genotype PCR forward primer binding at Chr2A: 5,510..5,529 |
| Chr2Del_F2 | TCAGCAAAATGATCGAGGCA | Genotype PCR forward primer binding at Chr2A: 274,792..274,811 |
| Chr2Del_R1 | AATGGAGCGTCAGCTAAAGG | Genotype PCR reverse primer binding at Chr2A: 275,578..275,559 |
| Chr2Del_R2 | AGCGATTGCTGGTCTCAAAA | Genotype PCR reverse primer binding at Chr2A: 276,530..276,511 |
| Chr2Del_R3 | CACCATGACATTCGGTGCTA | Genotype PCR reverse primer binding at Chr2A: 591,190..591,171 |
| SNR52_R_550 | acactggctctttttgatgaCAAATTAAAAATAGTTTACGCAAGTC | sgRNA synthesis against *C2_00550W* |
| sgRNA_F_550 | tcatcaaaaagagccagtgtGTTTTAGAGCTAGAAATAGCAAGTTAAA | sgRNA synthesis against *C2_00550W* |
| 550_NAT_FLP_Rev | cagccaccgccaaagcatccaagttagttgtcacatgattggtgactctatttgatagaatattgtgtatttcacctttaCTCTAGAACTAGTGGATCTG | Deletion construct with a homology arm to *C2_00550W* |
| SNR52_R_560 | acttcttcctcgatagcatcCAAATTAAAAATAGTTTACGCAAGTC | sgRNA synthesis against *C2_00560W* |
| sgRNA_F_560 | gatgctatcgaggaagaagtGTTTTAGAGCTAGAAATAGCAAGTTAAA | sgRNA synthesis against *C2_00560W* |
| 560_NAT_FLP_For | aaactattacgttaaatctgataatgaacaattcaaatgattggagtttttcattatgtgatgactctcttgaactatttTAAAGGGAACAAAAGCTGGG | Deletion construct with a homology arm to *C2_00560W* |
| SNR52_R_1140 | atatggacttctatgttgaaCAAATTAAAAATAGTTTACGCAAGTC | sgRNA synthesis against *C2_01140C* |
| sgRNA_F_1140 | ttcaacatagaagtccatatGTTTTAGAGCTAGAAATAGCAAGTTAAA | sgRNA synthesis against *C2_01140C* |
| 1140_NAT_FLP_Rev | ttgtattcatattgagtcccatcttttgcaaccaacacaggcatattcttgtacgtcagtgaattagggatgaaagggacCTCTAGAACTAGTGGATCTG | Deletion construct with a homology arm to *C2_01140C* |
| SNR52_R_1150 | cggtacttgttacaattaaaCAAATTAAAAATAGTTTACGCAAGTC | sgRNA synthesis against *C2_01150W* |
| sgRNA_F_1150 | tttaattgtaacaagtaccgGTTTTAGAGCTAGAAATAGCAAGTTAAA | sgRNA synthesis against *C2_01150W* |
| 1150_NAT_FLP_For | agctaatatctgcaaatcaattagagtcattattatctccatcattacgatacattttggttcactatgccagtaaatatTAAAGGGAACAAAAGCTGGG | Deletion construct with a homology arm to *C2_01150W* |
| Chr2Del_F3 | CGAACTTGACGACCTAGACG | Genotype PCR forward primer binding at Chr2A: 6,351..6,370 |
| Chr2Del_R4 | TCGTCACAGAACTTTAGCGG | Genotype PCR reverse primer binding at Chr2A: 7,055..7,036 |
| Chr2Del_R5 | AAGAGGACCAACCAGTACCA | Genotype PCR reverse primer binding at Chr2A: 88,739..88,720 |
| Chr2Del_F4 | GCCCTGCAAGTATCCAATCA | Genotype PCR forward primer binding at Chr2A: 89,570..89,589 |
| Chr2Del_F5 | CAACCTCTCCCCAAACTCAC | Genotype PCR forward primer binding at Chr2A: 90,395..90,414 |
| Chr2Del_R7 | GAGGTTCTGGTTCTTCGGTG | Genotype PCR reverse primer binding at Chr2A: 91,229..91,210 |
| Chr2Del_R6 | TTGGGGTTGTTTTCCGACAT | Genotype PCR reverse primer binding at Chr2A: 185,045..185,064 |
| Chr2Del_F6 | AACTCAGTCATTGGACACGC | Genotype PCR forward primer binding at Chr2A: 186,488..186,507 |
| 1140_NAT_FLP_Rev | atcaatgcaaagcttgaaactgattattgaaactaataaaatcaggccagcaaaacttaaggagtttgatttgatatttaCTCTAGAACTAGTGGATCTG | Deletion construct with a homology arm to *C2_01140C* |
| Chr2Del_R8 | TTCTGTGAATGTCCCTTGCC | Genotype PCR reverse primer binding at Chr2A: 186887..186868 |
| 1300_NAT_FLP_Rev | atcccagtatcatcaaaaactgttcgtcttatacttgtgtcattacttctaataacactaataaatatattggctgctttCTCTAGAACTAGTGGATCTG | Deletion construct with a homology arm to *C2_01300C* |
| SNR52_R_1300 | acggtcaactttatcatcatCAAATTAAAAATAGTTTACGCAAGTC | sgRNA synthesis against *C2_01300C* |
| sgRNA_F_1300 | atgatgataaagttgaccgtGTTTTAGAGCTAGAAATAGCAAGTTAAA | sgRNA synthesis against *C2_01300C* |
| 1310_NAT_FLP_For | catcaccaacaacgaaaatagttgttaaaagacgatatagtgaatttaaatctttgagagacaatttattaaaattattcTAAAGGGAACAAAAGCTGGG | Deletion construct with a homology arm to *C2_01310W* |
| SNR52_R_1310 | acattaataatcccacccatCAAATTAAAAATAGTTTACGCAAGTC | sgRNA synthesis against *C2_01310W* |
| sgRNA_F_1310 | atgggtgggattattaatgtGTTTTAGAGCTAGAAATAGCAAGTTAAA | sgRNA synthesis against *C2_01310W* |
| 1420_NAT_FLP_Rev | agtatgatcagaatattagtgattcagaacacgatttaacaccaatcaaaagaaagcgtcaatcagcacaatcggcaccaCTCTAGAACTAGTGGATCTG | Deletion construct with a homology arm to *C2_01420C* |
| SNR52_R_1420 | ccgccagcaacaaagttttcCAAATTAAAAATAGTTTACGCAAGTC | sgRNA synthesis against *C2_01420C* |
| sgRNA_F_1420 | gaaaactttgttgctggcggGTTTTAGAGCTAGAAATAGCAAGTTAAA | sgRNA synthesis against *C2_01420C* |
| 1430_NAT_FLP_For | ctaacttcccaaaagaaggaattttatttgaagatttcttaccaattttcactaagccagacttgtttaataaattagtcTAAAGGGAACAAAAGCTGGG | Deletion construct with a homology arm to *C2_01430W* |
| SNR52_R_1430 | acgtgtaatttgaaagctttCAAATTAAAAATAGTTTACGCAAGTC | sgRNA synthesis against *C2_01430W* |
| sgRNA_F_1430 | aaagctttcaaattacacgtGTTTTAGAGCTAGAAATAGCAAGTTAAA | sgRNA synthesis against *C2_01430W* |
| Chr2Del_R9 | TTGCTCGTCCCCTTTCATAC | Genotype PCR reverse primer binding at Chr2A: 221,879..221,860 |
| SNR52_R_1420_new | TAGCATAGACTATATCTGTTCAAATTAAAAATAGTTTACGCAAGTC | sgRNA synthesis against *C2_01420C* |
| sgRNA_F_1420_new | AACAGATATAGTCTATGCTAGTTTTAGAGCTAGAAATAGCAAGTTAAA | sgRNA synthesis against *C2_01420C* |
| 1420_NAT_Rev_new | AAGTTTTACTCTTGAACAATCACCAGAAATAAAACCTAAACCTAAATCAAAAACTTCAGATTTAACAGATATAGTCTATGCTCTAGAACTAGTGGATCTG | Deletion construct with a homology arm to *C2_01420C* |
| Chr2Del_R10 | GTACAGACCGACACAACTCC | Genotype PCR reverse primer binding at Chr2A: 256,183..256,164 |
| SNR52_R_1460 | AGCACTTTTTAATATTTAACCAAATTAAAAATAGTTTACGCAAGTC | sgRNA synthesis against *C2_01460C* |
| sgRNA_F_1460 | GTTAAATATTAAAAAGTGCTGTTTTAGAGCTAGAAATAGCAAGTTAAA | sgRNA synthesis against *C2_01460C* |
| 1460_NAT_FLP_Rev | GCAACACTTTAACAAAAAGTGTATTCATGTTCTATAAGAACAATTGAGAAGACAGAGTATAAACCCAACATTGCTTACAACTCTAGAACTAGTGGATCTG | Deletion construct with a homology arm to *C2_01460C* |
| Chr2Del_R11 | AACCTAGCATTGATGGAGCC | Genotype PCR reverse primer binding at Chr2A: 260,529..260,510 |
| SNR52_R_1500 | TTGGCATATATTGAAATACACAAATTAAAAATAGTTTACGCAAGTC | sgRNA synthesis against *C2_01500W* |
| sgRNA_F_1500 | TGTATTTCAATATATGCCAAGTTTTAGAGCTAGAAATAGCAAGTTAAA | sgRNA synthesis against *C2_01500W* |
| 1500_NAT_FLP_Rev | TTCTGGTGAGTACGTAGAGAGTAATTGAGCAACAAGATCCTAAAAAAAAAAGCGTAGTCTAGTAGAGAAAAACAATTCAGCTCTAGAACTAGTGGATCTG | Deletion construct with a homology arm to *C2_01500W* |
| Chr2Del_R12 | ACAAAATGAGTCGGCACCAA | Genotype PCR reverse primer binding at Chr2A: 271,047..271,028 |
| 1470_NAT_FLP_For | TTTAACTTCAGCTTTCTTCTGGCTACTACAATCTTCACTTACAACTAGAATTCATACTAAAAACATTCGATAGAAACATCTAAAGGGAACAAAAGCTGGG | Deletion construct with a homology arm to *C2_01470W* |
| 1470_NAT_FLP_Rev | AAGTCGTGCGAAATGGCCATACTCATTAAACCTATGTACAGCTTTCAATCTTTATCTACATATATGTGCACTAAATAATCCTCTAGAACTAGTGGATCTG | Deletion construct with a homology arm to *C2_01470W* |
| 1480_NAT_FLP_For | AACAGAGATCTCTGGTCATTAATATTCACAGCAATTTACACATCATCTCAACTATATCAAATGTCAGACAGTGATCTACCTAAAGGGAACAAAAGCTGGG | Deletion construct with a homology arm to *C2_01480W* |
| 1480_NAT_FLP_Rev | ATCAACATAAATAAACAAAGACAGTAGTGAAGAACTAACAAGGGAGCGCACTTCAGTTAGAAAAACAACATTTCTTGGTACTCTAGAACTAGTGGATCTG | Deletion construct with a homology arm to *C2_01480W* |
| 1490_NAT_FLP_For | AATACCAAATATAATTTTAATTGTTTTCACAACCTGGACAATTATGAAGAACTTCGTGCACAAAAACATCACAGTTGATATAAAGGGAACAAAAGCTGGG | Deletion construct with a homology arm to *C2_01490C* |
| 1490_NAT_FLP_Rev | TGGGCTCTAAATCGCCGTCCTCTAAACTTCCTTCGACTGCTCCAGAGAAAAGTGCATCACCTGGAGGAGCAAAGAGTGGTCTCTAGAACTAGTGGATCTG | Deletion construct with a homology arm to *C2_01490C* |
| SNR52_R_770 | CAATGTCTAAAGCTTTATCTCAAATTAAAAATAGTTTACGCAAGTC | sgRNA synthesis against *C2_00770W* |
| sgRNA_F_770 | AGATAAAGCTTTAGACATTGGTTTTAGAGCTAGAAATAGCAAGTTAAA | sgRNA synthesis against *C2_00770W* |
| Chr2Del_F7 | GAGAGTTAACGGCGTGACTT | Genotype PCR forward primer binding at Chr2A: 131,649..131,668 |
| 770_NAT_FLP_For | ACTACACCACGCTACACATTTTCTTTTATACCCCTTGAATTAACAATTCTATTTCAAACAAATACTTATAATCTTCATAATAAAGGGAACAAAAGCTGGG | Deletion construct with a homology arm to *C2_00770W* |
| SNR52_R_1000 | CAGCTGTAGATGTTGATGAGCAAATTAAAAATAGTTTACGCAAGTC | sgRNA synthesis against *C2_01000W* |
| sgRNA_F_1000 | CTCATCAACATCTACAGCTGGTTTTAGAGCTAGAAATAGCAAGTTAAA | sgRNA synthesis against *C2_01000W* |
| Chr2Del_F8 | ACACAATCACCCTACACTGC | Genotype PCR forward primer binding at Chr2A: 163,901..163,920 |
| 1000_NAT_FLP_For | CCGAGTTTAGTTTCATTTTTCCTTTTTCTCTTTTTCTACATCATCCTCACAACAATTTCAAATATGTCTCAAGACAACGTTAAAGGGAACAAAAGCTGGG | Deletion construct with a homology arm to *C2_01000W* |
| 1470_check_For | AGTGAAGTAGGAGACACACGA | Genotype PCR primer for detecting *C2_01470W* allele |
| 1470_check_Rev | TCGACCTTGTTGTGACCAAA | Genotype PCR primer for detecting *C2_01470W* allele |
| 1480_check_For | GGTATTGACCCTGAGGTGGT | Genotype PCR primer for detecting *C2_01480W* allele |
| 1480_check_Rev | TTGATCACCGGCTCTTCTGA | Genotype PCR primer for detecting *C2_01480W* allele |
| 1490_check_For | CTGCTTGGAACGGAGGTATG | Genotype PCR primer for detecting *C2_01490C* allele |
| 1490_check_Rev | AGCCCAATATACGACAGCAC | Genotype PCR primer for detecting *C2_01490C* allele |
| BCY1_For | TGTGATACCGGTCTTTTAGCA | Genotype PCR primer for detecting *BCY1* allele |
| BCY1_Rev | CACCAGAAGGTGAGTAAGCC | Genotype PCR primer for detecting *BCY1* allele |
| BCY1_NAT_FLP_For | ATGTTGCCCACAAATAAATTGTGATACCGGTCTTTTAGCATATATCTTCTACTCTTCAATCAACATCTTTACCAATGTCTTAAAGGGAACAAAAGCTGGG | *BCY1* deletion construct |
| BCY1_NAT_FLP_Rev | GGTGAGTAAGCCGAAAAAAAAAAGATAGAATATACTAACATAAAACAATATAAGATAATAAACAACTACTTATTGTACACCTCTAGAACTAGTGGATCTG | *BCY1* deletion construct |
| sgMOB2_top | atttgTTTTGAAGATAATTTTGAAGg | CRISPR plasmid cloning against *MOB2* |
| sgMOB2_bottom | aaaacCTTCAAAATTATCTTCAAAAc | CRISPR plasmid cloning against *MOB2* |
| MOB2_S49A_For | ATCTGGCAATGGTTTGAGACGAACACAATCACCTACCAAGTTTgCgCCaTCAAAATTATC | Repair template synthesis for *MOB2* S49A mutation |
| MOB2_S49A_Rev | AGAAGACGTGTATGCAGCTGAGCCTTGTGCACCTTTTGAAGATAATTTTGAtGGcGcAAA | Repair template synthesis for *MOB2* S49A mutation |
| MOB2_check_For | GGCGGGTATCCATTAAGCAA | Genotype PCR primer for detecting *MOB2* allele |
| MOB2_check_Rev | GTTTGGTCCCGCATTCATTG | Genotype PCR primer for detecting *MOB2* allele |
| sgBNI1_top | atttgAAAAGGACAACTGGGTCTGTg | CRISPR plasmid cloning against *BNI1* |
| sgBNI1_bottom | aaaacACAGACCCAGTTGTCCTTTTc | CRISPR plasmid cloning against *BNI1* |
| BNI1_S1619A_For | TTAATGAGAAAACAAATATTGGAAAGTCAACGTAAAAGaACtACaGGaTCgGTTGGCTCA | Repair template synthesis for *BNI1* S1619A mutation |
| BNI1_S1619A_Rev | TGTCAGATTCATTATTTCTTGTtGGcGcGACATTAGTTGGTGAGCCAACcGAtCCtGTaG | Repair template synthesis for *BNI1* S1619A mutation |
| BNI1_check_For | ACTGAGTCGATCCGTACCAT | Genotype PCR primer for detecting *BNI1* allele |
| BNI1_check_Rev | TTTATGTGTCGCCATCGTCA | Genotype PCR primer for detecting *BNI1* allele |
| SNR52_R_HGC1 | AGTTCATCATGTAGTACTACCAAATTAAAAATAGTTTACGCAAGTC | sgRNA synthesis against *HGC1* |
| sgRNA_F_HGC1 | GTAGTACTACATGATGAACTGTTTTAGAGCTAGAAATAGCAAGTTAAA | sgRNA synthesis against *HGC1* |
| HGC1_check_For | TGTGCGTTGTGCGTGTATAA | Genotype PCR primer for detecting *HGC1* allele |
| HGC1_check_Rev | GGTGAAAGTTAAATATGGTTGTTGT | Genotype PCR primer for detecting *HGC1* allele |
| HGC1_NAT_FLP_For | CATCAAAGCATCAAACCAATACCCAACACTTTAATATCTAGGGTTTCCATTCACATATACACATATAAACATATATTAATTAAAGGGAACAAAAGCTGGG | *HGC1* deletion construct |
| HGC1_NAT_FLP_Rev | CATCATTAAAATTTCATATCATAATAACAACATCTTTCTCCATTCTCCATTCTCTACTTTATCTTTCTCTCTTTCTTTAACTCTAGAACTAGTGGATCTG | *HGC1* deletion construct |
| SNR52_R_YCK2 | TCACATAATGTAACTGCTGGCAAATTAAAAATAGTTTACGCAAGTC | sgRNA synthesis against *YCK2* |
| sgRNA_F_YCK2 | CCAGCAGTTACATTATGTGAGTTTTAGAGCTAGAAATAGCAAGTTAAA | sgRNA synthesis against *YCK2* |
| YCK2_check_For | AATGCTACTTGGCTAATCCCA | Genotype PCR primer for detecting *YCK2* allele |
| YCK2_check_Rev | AGGAATTCCGTCACATCCTTG | Genotype PCR primer for detecting *YCK2* allele |
| YCK2_NAT_FLP_For | CGTTGAGACtTGACAACAAACCCTGCTTTGGCGGCTGCTCAAGCATCTCATAATAATATTCCTACAAAGCAAATGAATCATAAAGGGAACAAAAGCTGGG | *YCK2* deletion construct |
| YCK2_NAT_FLP_Rev | GATATCATACAACTAATGACAACACAATTTAGACCAGAACCCTTTGTTTTCTTCTTCTTCGGCAACCATTTGTTGTTGTTCTCTAGAACTAGTGGATCTG | *YCK2* deletion construct |
| HGC1_check_Rev2 | GGTGGTGATGGTCTGTTCAT | Genotype PCR primer for detecting *HGC1* allele |
