# Supplementary material for: Integrative multi-omics profiling reveals cAMP-independent mechanisms regulating hyphal morphogenesis in Candida albicans
Source: PLoS Pathog. 2021 Aug 16;17(8):e1009861. doi: 10.1371/journal.ppat.1009861 (PMC8389844; doi:10.1371/journal.ppat.1009861)
Supplement: S1 Text — (DOCX) [file ppat.1009861.s016.docx]

**SUPPLEMENTAL METHODS**

**1. Protocol for transient CRISPR-Cas9 system**

The transient CRISPR system requires CaCas9 expression cassette, sgRNA expression cassette, and target gene deletion construct. The three components were all PCR amplified as described below.

**1.1. Construction of CaCas9 expression cassette**

The CaCas9 expression cassette was PCR amplified from the plasmid pV1093. The plasmid pV1093 used in this study was a kind gift from Valmik Vyas [Sci Adv 1(3):e1500248, 2015].

| Reagent | Volume (µl) |
| --- | --- |
| pV1093 (50 ng/ µl) | 1.0 |
| 10X buffer | 5.0 |
| dNTP (2.5 mM each) | 4.0 |
| 10µM CaCas9/For primer | 1.0 |
| 10µM CaCas9/Rev primer | 1.0 |
| TAKARA ExTaq | 0.25 |
| Sterilized deionized water | Up to 50.0 |

| Temperature | Time | No. of cycles |
| --- | --- | --- |
| 94 °C | 1 min |  |
| 94 °C | 30 sec | 30 cycles |
| 58 °C | 1 min |  |
| 72 °C | 4 min |  |
| 72 °C | 5 min |  |
| 4 °C | - |  |

Purify the PCR products and measure the concentration.

**1.2. Construction of sgRNA expression cassette**


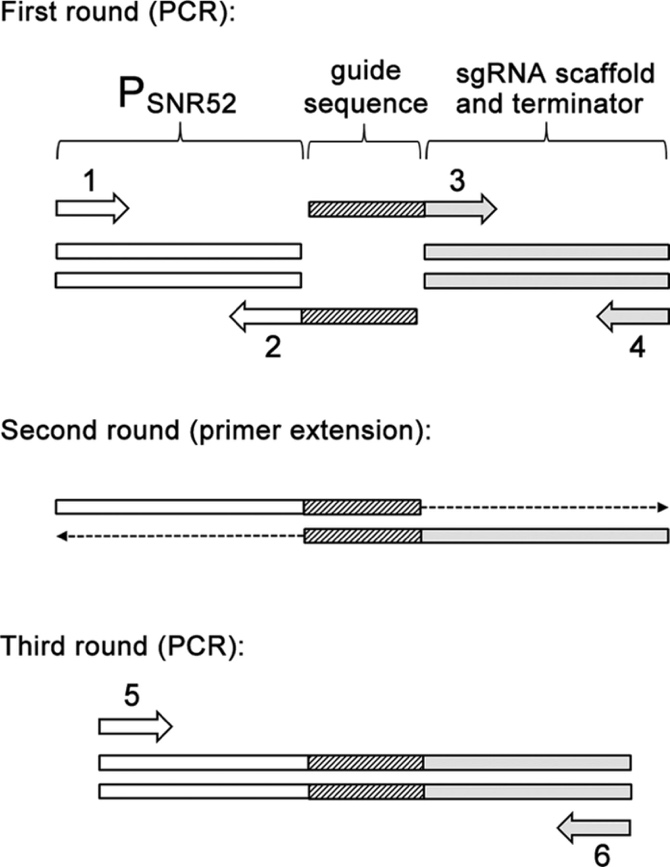


Three DNA synthesis steps fuse DNA fragments comprising the SNR52 promoter and the sgRNA scaffold. Chimeric primers 2 and 3 carry 20 complementary bases of guide sequence. The guide sequence was designed and used by Vyas et al. [Sci Adv 1(3):e1500248, 2015].

In the first step, PCR is used to create two segments of the sgRNA gene. The SNR52 promoter is amplified with primers 1 and 2; the sgRNA scaffold is amplified with primers 3 and 4. In the second step, primer extension is used to fuse the two PCR products, with chimeric extensions acting as primers. In the third step, PCR with nested primers 5 and 6 is used to amplify the final sgRNA expression cassette.

**First round PCR**

SNR52 promoter region

| Reagent | Volume (µl) |
| --- | --- |
| pV1093 (50 ng/ µl) | 1.0 |
| 10X buffer | 5.0 |
| dNTP (2.5 mM each) | 4.0 |
| 10µM primer #1 (SNR52/F) | 1.0 |
| 10µM primer #2 (SNR52/R) | 1.0 |
| TAKARA ExTaq | 0.25 |
| Sterilized deionized water | Up to 50.0 |

sgRNA scaffold region

| Reagent | Volume (µl) |
| --- | --- |
| pV1093 (50 ng/ µl) | 1.0 |
| 10X buffer | 5.0 |
| dNTP (2.5 mM each) | 4.0 |
| 10µM primer #3 (sgRNA/F) | 1.0 |
| 10µM primer #4 (sgRNA/R) | 1.0 |
| TAKARA ExTaq | 0.25 |
| Sterilized deionized water | Up to 50.0 |

| Temperature | Time | No. of cycles |
| --- | --- | --- |
| 94 °C | 1 min |  |
| 94 °C | 30 sec | 30 cycles |
| 58 °C | 1 min |  |
| 72 °C | 1 min |  |
| 72 °C | 5 min |  |
| 4 °C | - |  |

Purify the PCR products and measure the concentration.

**Second round PCR**

| Reagent | Volume (µl) |
| --- | --- |
| Purified SNR52 promoter amplicon | 2.5 |
| Purified sgRNA amplicon | 2.5 |
| 10X buffer | 2.5 |
| dNTP (2.5 mM each) | 2.0 |
| TAKARA ExTaq | 0.25 |
| Sterilized deionized water | Up to 25.0 |

*Note. Use 1: 1 molar ratio for SNR52: sgRNA amplicons. The total DNA amount of the two components should be between 100 and 1000 ng.*

| Temperature | Time | No. of cycles |
| --- | --- | --- |
| 94 °C | 2 min |  |
| 94 °C | 30 sec | 10 cycles |
| 58 °C | 10 min |  |
| 72 °C | 5 min |  |
| 72 °C | 10 min |  |
| 4 °C | - |  |

Do not need to purify the PCR products and measure the concentration.

**Third round PCR**

| Reagent | Volume (µl) |
| --- | --- |
| Second round product | 1.0 |
| 10X buffer | 5.0 |
| dNTP (2.5 mM each) | 4.0 |
| 10µM primer #5 (SNR52/N) | 1.0 |
| 10µM primer #6 (sgRNA/N) | 1.0 |
| TAKARA ExTaq | 0.25 |
| Sterilized deionized water | Up to 50.0 |

| Temperature | Time | No. of cycles |
| --- | --- | --- |
| 94 °C | 1 min |  |
| 94 °C | 30 sec | 30 cycles |
| 58 °C | 1 min |  |
| 72 °C | 1 min |  |
| 72 °C | 5 min |  |
| 4 °C | - |  |

Purify the PCR products and measure the concentration.

**1.3. Construction of gene deletion constructs**

Gene deletion constructs were synthesized by PCR using the plasmid pGR-NAT, as a template. The primers were designed to include 80 bases with homology to the sequences upstream or downstream from the target gene.

| Reagent | Volume (µl) |
| --- | --- |
| Template plasmid (50 ng/µl) | 1.0 |
| 10X buffer | 5.0 |
| dNTP (2.5 mM each) | 4.0 |
| 10µM Forward primer | 1.0 |
| 10µM Reverse primer | 1.0 |
| TAKARA ExTaq | 0.25 |
| Sterilized deionized water | Up to 50.0 |

| Temperature | Time | No. of cycles |
| --- | --- | --- |
| 94 °C | 1 min |  |
| 94 °C | 30 sec | 30 cycles |
| 58 °C | 1 min |  |
| 72 °C | 2 min |  |
| 72 °C | 5 min |  |
| 4 °C | - |  |

Purify the PCR products and measure the concentration.

**1.4. Fungal transformation**

PCR products for transformation were purified and concentrated with the commercial PCR purification kit. In the transient CRISPR system, the deletion constructs (1 ug) were co-transformed with the *CaCAS9* cassette (1 ug) and sgRNA cassette (1 ug), using the electroporation method.

**1.5. Primers**

| **Primer** | **Sequence** | **Description** |
| --- | --- | --- |
| CaCas9/for | ATCTCATTAGATTTGGAACTTGTGGGTT | Forward and reverse primers for amplification of *CaCas9* cassette |
| CaCas9/rev | TTCGAGCGTCCCAAAACCTTCT |  |
| SNR52/F | AAGAAAGAAAGAAAACCAGGAGTGAA | Forward primer for amplification of *SNR52* promoter |
| SNR52/R | (Reverse complementary of 20-nt target) + CAAATTAAAAATAGTTTACGCAAGTC | Reverse primer for amplification of *SNR52* promoter with overlapping guide sequence |
| sgRNA/F | (20-nt target sequence) + GTTTTAGAGCTAGAAATAGCAAGTTAAA | Forward primer for amplification of sgRNA scaffold with overlapping guide sequence |
| sgRNA/R | ACAAATATTTAAACTCGGGACCTGG | Reverse primer for amplification of sgRNA scaffold |
| SNR52/N | GCGGCCGCAAGTGATTAGACT | Forward and reverse nested primers for third round PCR for construction of sgRNA expression cassette |
| sgRNA/N | GCAGCTCAGTGATTAAGAGTAAAGATGG |  |

**1.6. References**

Min K, Ichikawa Y, Woolford CA, Mitchell AP. 2016. *Candida albicans* gene deletion with a transient CRISPR-Cas9 system. *mSphere* **1**(3):e00130-16. doi:10.1128/mSphere.00130-16

Vyas VK, Barrasa MI, Fink GR. 2015. A *Candida albicans* CRISPR system permits genetic engineering of essential genes and gene families. *Sci Adv* **1**:e1500248. doi:10.1126/sciadv.1500248.

Yu JH, Hamari Z, Han KH, Seo JA, Reyes-Domínguez Y, Scazzocchio C. 2004. Double-joint PCR: a PCR-based molecular tool for gene manipulations in filamentous fungi. *Fungal Genet Biol* **41**:973–981. doi:10.1016/j.fgb.2004.08.001.

**2. Large CRISPR-mediated deletion**

**KM11** (*bcy1∆*/*BCY1 cyr1∆*/*∆*): We first made the KM11 strain without using the CRISPR system. The parental *cyr1∆*/*∆* strain was made in the previous study (Parrino et al., 2017) and the NAT selection marker (SAT1-FLP) was excised for marker recycling. The *BCY1* deletion construct was synthesized with the BCY1_NAT_FLP_For and BCY1_NAT_FLP_Rev primers, using the plasmid pGR-NAT plasmid as a template. The construct was transformed into the *cyr1∆*/*∆* cells and the heterozygous *BCY1* deletion mutants were selected for NAT resistance. PCR genotyping of the transformants verified the heterozygous deletion. The NAT selection marker (SAT1-FLP) was excised again for large CRISPR-mediated deletion.

**KM12** (*Chr2L 270kb deletion*): When deleting 270kb region of chromosome 2 for gene mapping, two sgRNA expression cassettes were used to cut the C2_00030W and C2_01540W loci. Chimeric primers SNR52_R_30 and sgRNA_F_30 were used to synthesize the sgRNA cassette against C2_00030W. Chimeric primers SNR52_R_1540 and sgRNA_F_1540 were used to synthesize the sgRNA cassette against C2_01540W. The 270kb deletion construct was synthesized with the 30_NAT_FLP_For and 1540_NAT_FLP_Rev primers, using the plasmid pGR-NAT plasmid as a template. The *CaCAS9* expression cassette, two sgRNA expression cassettes, and deletion construct were co-transformed into the Nat^s^ KM11 strain. Nat^r^ transformants were selected, and PCR genotyping of the transformants verified the heterozygous deletion.

**KM13** (*Chr2L 590kb deletion*): When deleting 590kb region of chromosome 2 for gene mapping, two sgRNA expression cassettes were used to cut the C2_00030W and C2_02960C loci. Chimeric primers SNR52_R_30 and sgRNA_F_30 were used to synthesize the sgRNA cassette against C2_00030W. Chimeric primers SNR52_R_2960 and sgRNA_F_2960 were used to synthesize the sgRNA cassette against C2_02960C. The 590kb deletion construct was synthesized with the 30_NAT_FLP_For and 2960_NAT_FLP_Rev primers, using the plasmid pGR-NAT plasmid as a template. The *CaCAS9* expression cassette, two sgRNA expression cassettes, and deletion construct were co-transformed into the Nat^s^ KM11 strain. Nat^r^ transformants were selected, and PCR genotyping of the transformants verified the heterozygous deletion.

**KM14** (*Chr2L 90kb deletion*): When deleting 90kb region of chromosome 2 for gene mapping, two sgRNA expression cassettes were used to cut the C2_00030W and C2_00550W loci. Chimeric primers SNR52_R_30 and sgRNA_F_30 were used to synthesize the sgRNA cassette against C2_00030W. Chimeric primers SNR52_R_550 and sgRNA_F_550 were used to synthesize the sgRNA cassette against C2_00550W. The 90kb deletion construct was synthesized with the 30_NAT_FLP_For and 550_NAT_FLP_Rev primers, using the plasmid pGR-NAT plasmid as a template. The *CaCAS9* expression cassette, two sgRNA expression cassettes, and deletion construct were co-transformed into the Nat^s^ KM11 strain. Nat^r^ transformants were selected, and PCR genotyping of the transformants verified the heterozygous deletion.

**KM15** (*Chr2L 90kb→180kb deletion*): When deleting 90kb→180kb region of chromosome 2 for gene mapping, two sgRNA expression cassettes were used to cut the C2_00560W and C2_01140C loci. Chimeric primers SNR52_R_560 and sgRNA_F_560 were used to synthesize the sgRNA cassette against C2_00560W. Chimeric primers SNR52_R_1140 and sgRNA_F_1140 were used to synthesize the sgRNA cassette against C2_01140C. The 90kb→180kb deletion construct was synthesized with the 560_NAT_FLP_For and 1140_NAT_FLP_Rev primers, using the plasmid pGR-NAT plasmid as a template. The *CaCAS9* expression cassette, two sgRNA expression cassettes, and deletion construct were co-transformed into the Nat^s^ KM11 strain. Nat^r^ transformants were selected, and PCR genotyping of the transformants verified the heterozygous deletion.

**KM16** (*Chr2L 180kb→270kb deletion*): When deleting 180kb→270kb region of chromosome 2 for gene mapping, two sgRNA expression cassettes were used to cut the C2_01150W and C2_01540W loci. Chimeric primers SNR52_R_1150 and sgRNA_F_1150 were used to synthesize the sgRNA cassette against C2_1150W. Chimeric primers SNR52_R_1540 and sgRNA_F_1540 were used to synthesize the sgRNA cassette against C2_01540W. The 590kb deletion construct was synthesized with the 1150_NAT_FLP_For and 1540_NAT_FLP_Rev primers, using the plasmid pGR-NAT plasmid as a template. The *CaCAS9* expression cassette, two sgRNA expression cassettes, and deletion construct were co-transformed into the Nat^s^ KM11 strain. Nat^r^ transformants were selected, and PCR genotyping of the transformants verified the heterozygous deletion.

**KM17** (*Chr2L 180kb deletion*): When deleting 180kb region of chromosome 2 for gene mapping, two sgRNA expression cassettes were used to cut the C2_00030W and C2_01140C loci. Chimeric primers SNR52_R_30 and sgRNA_F_30 were used to synthesize the sgRNA cassette against C2_00030W. Chimeric primers SNR52_R_1140 and sgRNA_F_1140 were used to synthesize the sgRNA cassette against C2_01140C. The 180kb deletion construct was synthesized with the 30_NAT_FLP_For and 1140_NAT_FLP_Rev primers, using the plasmid pGR-NAT plasmid as a template. The *CaCAS9* expression cassette, two sgRNA expression cassettes, and deletion construct were co-transformed into the Nat^s^ KM11 strain. Nat^r^ transformants were selected, and PCR genotyping of the transformants verified the heterozygous deletion.

**KM18** (*Chr2L 90kb→270kb deletion*): When deleting 90kb→270kb region of chromosome 2 for gene mapping, two sgRNA expression cassettes were used to cut the C2_00560W and C2_01540W loci. Chimeric primers SNR52_R_560 and sgRNA_F_560 were used to synthesize the sgRNA cassette against C2_00560W. Chimeric primers SNR52_R_1540 and sgRNA_F_1540 were used to synthesize the sgRNA cassette against C2_01540W. The 90kb→270kb deletion construct was synthesized with the 560_NAT_FLP_For and 1540_NAT_FLP_Rev primers, using the plasmid pGR-NAT plasmid as a template. The *CaCAS9* expression cassette, two sgRNA expression cassettes, and deletion construct were co-transformed into the Nat^s^ KM11 strain. Nat^r^ transformants were selected, and PCR genotyping of the transformants verified the heterozygous deletion.

**KM19** (*Chr2L 90kb→260kb deletion*): When deleting 90kb→260kb region of chromosome 2 for gene mapping, two sgRNA expression cassettes were used to cut the C2_00560W and C2_01500W loci. Chimeric primers SNR52_R_560 and sgRNA_F_560 were used to synthesize the sgRNA cassette against C2_00560W. Chimeric primers SNR52_R_1500 and sgRNA_F_1500 were used to synthesize the sgRNA cassette against C2_01500W. The 90kb→260kb deletion construct was synthesized with the 560_NAT_FLP_For and 1500_NAT_FLP_Rev primers, using the plasmid pGR-NAT plasmid as a template. The *CaCAS9* expression cassette, two sgRNA expression cassettes, and deletion construct were co-transformed into the Nat^s^ KM11 strain. Nat^r^ transformants were selected, and PCR genotyping of the transformants verified the heterozygous deletion.

**KM20** (*Chr2L 90kb→250kb deletion*): When deleting 90kb→250kb region of chromosome 2 for gene mapping, two sgRNA expression cassettes were used to cut the C2_00560W and C2_01460C loci. Chimeric primers SNR52_R_560 and sgRNA_F_560 were used to synthesize the sgRNA cassette against C2_00560W. Chimeric primers SNR52_R_1460 and sgRNA_F_1460 were used to synthesize the sgRNA cassette against C2_01460C. The 90kb→250kb deletion construct was synthesized with the 560_NAT_FLP_For and 1460_NAT_FLP_Rev primers, using the plasmid pGR-NAT plasmid as a template. The *CaCAS9* expression cassette, two sgRNA expression cassettes, and deletion construct were co-transformed into the Nat^s^ KM11 strain. Nat^r^ transformants were selected, and PCR genotyping of the transformants verified the heterozygous deletion.
