# Supplementary material for: Integrative multi-omics profiling reveals cAMP-independent mechanisms regulating hyphal morphogenesis in Candida albicans
Source: PLoS Pathog. 2021 Aug 16;17(8):e1009861. doi: 10.1371/journal.ppat.1009861 (PMC8389844; doi:10.1371/journal.ppat.1009861)
Supplement: Attachment — Submitted filename: ResponseToReviewers_Min_June2021.docx [file ppat.1009861.s017.docx]

**Response to Reviewers**

We thank the reviewers for their careful reading of our manuscript and their thoughtful comments that have helped us to improve the text and the presentation of the data.

**Part I - Summary**
Please use this section to discuss strengths/weaknesses of study, novelty/significance, general execution and scholarship.

**Reviewer #1:** For many years the cyr1 adenylate cyclase has been thought to play a critical role in promoting C. albicans hyphal morphogenesis, an important virulence trait of this major human fungal pathogen. In this study the authors characterize pseudorevertants of the cyr1 mutant which are capable of forming filaments in the absence of cAMP. Using genomics they show that chromosome 2 haploinsufficiency at BCY1, a negative regulatory subunit of PKA can rescue the slow growth defect of the cyr1 mutant and an additional multigenic haploinsufficiency at SRB9 and STP5, two global transcriptional regulators, can rescue the filamentation defect of the cyr1 mutant. Integrating information from transcriptomics, proteomics and phophoproteomics revealed that Cdc28 and Yck1 phosphorylation activities are active during filamentation of the pseudorevertants and that several transcriptional regulators of hyphal growth appear to be post-transcriptionally induced.

Overall, the manuscript is well-written and the data are clearly presented. This study is significant because it provides new information about non-cAMP-dependent mechanisms that drive C. albicans morphogenesis. In addition, the multi-omics data highlight the importance of post-transcriptional mechanisms for this morphological transition. However, there are several weaknesses as well. In particular, the manuscript could be strengthened by additional experiments, based on the multi-omics data, that provide new mechanistic information about the yeast-hyphal transition.

We revised the manuscript and added more quantitative data as described below.

**Reviewer #2**: In the fungal pathogen Candida albicans, adenylyl cyclase (Cyr1), which catalyzes cAMP synthesis, has long been thought to be essential for hyphal morphogenesis. cAMP activates protein kinase A (PKA) to trigger hyphal development. Several years back, James Konopka’s group isolated cyr1Δ/Δ pseudorevertant (PR) mutants that could form hyphae in the absence of cAMP. The finding was fascinating because it seemed to challenge a well-established model of C. albicans hyphal growth. However, how these PR mutants respond to induction to activate hyphal development was unknown.

As a follow-up study here, the Konopka group isolated additional PR mutants and conducted a comprehensive investigation of the molecular basis for the incredible hyphal growth in the PR mutants. First, they discovered that all the PR mutants shared one common genetic change, albeit by different means: the loss of one copy of BCY1, the negative regulatory subunit of protein kinase (A) from the left arm of chromosome 2. Furthermore, while a point mutation in one copy of BCY1 moderately restored hyphal morphogenesis, multigenic haploinsufficiency resulting from loss of large regions of the left arm of chromosome 2 further improved hyphal growth. Using CRISPR/cas9-mediated truncation of the left arm of Chr 2, they identified a 10-kb region important for improving hyphal growth. Interestingly, this region includes global transcriptional regulators. By RNA-Seq transcriptomic analysis, the authors found that many hyphal-associated genes were induced in the absence of cAMP. This indicates that basal PKA activity is an important prerequisite to induce hyphae, but activation of the cAMP pathway is not needed. The phosphoproteomic analysis further indicated that the cyclin-dependent Cdc28 kinase and the casein kinase Yck2 play vital roles required for the hyphal growth in the PR mutants. Finally, integrating transcriptomic and proteomic data reveals that hyphal stimuli induce increased production of key transcription factors that contribute to polarized morphogenesis.

The significance of this study is multi-fold:
1. It demonstrates the fascinating genome plasticity of C. albicans in adaptation to harmful mutations to improve fitness (growth and hyphal development).
2. It indicates the presence of yet unknown signaling pathway that activates the hyphal development in the absence of cAMP.
3. Differential translation of transcription factors could be an important mechanism that contributes to hyphal growth.
The findings presented here open up exciting opportunities for future investigations.

I suggest that the authors modify some of their statements, which are sweeping and overly assertive.

We have extensively revised the manuscript to address the reviewer’s comments. We generally agree with the reviewer, and can see now that many of these issues stem from how one thinks of cAMP signaling. We now try to make it clearer when we are referring to the specific role of cAMP, versus the whole pathway that includes PKA etc.

Reviewer #3: The manuscript by Min et al. provides a stimulating analysis of how Candida albicans strains that are deficient in cAMP signaling are still able to undergo hyphal formation in response to certain environmental cues. This is a very important area of research given that filamentation is critical for C. albicans to be pathogenic in multiple infection models. The work is generally of high quality and contains a number of interesting observations and complementary experimental techniques. The fact that hyphae formation (and response to environmental cues) can occur without cAMP/CYR1 continues to be intriguing and indicates much still remains to be learnt about mechanisms of filamentation in this species. Overall, there was much to appreciate in the paper, although I had a number of questions / concerns that, if addressed, would substantially strengthen the paper.

Specific changes are described in detail below.

**Part II – Major Issues: Key Experiments Required for Acceptance**

**Reviewer #1:**

1. The multi-omics experiments are not accompanied by additional experiments providing independent experimental validation (eg: qRT-PCR and Westerns). These experiments are important to include as controls.

We note that we included Western blots of Bcy1 and PKA in Figure S2. These results were important to support the conclusions from the genomic and transcriptomic studies that loss of one copy of BCY from the PR strains leads to a lower level of Bcy1 production. However, the other results of the Transcriptomic studies were not further validated since they were based on the behavior of a large number of hyphal-induced genes that reinforce the results that PR mutants showed similar transcriptional responses as did the wild type cells. With recent improvements in library construction and RNA-seq depth we have more confidence in these results than older studies might have been able to provide. Also, independent RNA-seq studies were highly reproducible (S4A Fig.). Similarly, the proteomic studies were highly reproducible and the trends we report were not based on just one protein or peptide (Fig. 4).

2. Hgc1 and Yck1 have previously been shown to play important roles in C. albicans filamentous growth. The manuscript could be significantly strengthened by the demonstration that novel non-cAMP-dependent mechanisms elucidated from the multi-omics data play important roles in driving the C. albicans yeast-filament transition.

We revised the manuscript, including improvements to the summary Figure 6, to make it clearer that we have also identified roles for genome adaptation in response to *cyr1*Δ including novel roles for mediator and general transcription factors, and the regulation of hyphal transcription factors. In addition, we report surprisingly normal transcriptional responses to hyphal stimuli in the absence of adenylyl cyclase, and similar profiles in proteomic and phosphoproteomic analyses. These results show that although adenylyl cyclase is a major regulator of hyphal responses, additional pathways contribute to hyphal regulation.

**Reviewer #2:**

Page 6, the first paragraph. Using percentages alone to describe the ability of hyphal growth of the PR mutants can be misleading because it cannot accurately reflect the quality of hyphal growth. As shown in Figure 1B, most of the PR mutants show abnormal hyphal morphology. They are significantly shorter and thicker than WT hyphae. When you say 93% of PR18 cells form hyphae, it is not what the image shows. It is acceptable to say 93% of the cells respond to hyphal induction. The authors should modify the description.

We agree with the reviewer. We revised the manuscript to indicate that we are reporting the percent of filamentous cells. We also modified the text to describe more about the hyphal morphology. Please see page 6, the first paragraph. (We also note that the relative increase in the percent hyphal growth is likely an underrepresentation of the overall increase in filamentous growth. We had an unbiased person quantify the percent of filamentous cells. They counted filamentous cells but did not distinguish based on the changes in hyphal width or length.)

**Reviewer #3:**

1. One question concerned the emergence of mutants and their placement in different classes. The authors classify the cyr1 revertants into 4 classes (1, 2, 3 and 3+). It was not clear how many PR revertants were analyzed and which ones fall into each class. Also, were truly independent revertants identified for each class? For example, it was striking that the two class 2 revertants appear to have the same genomic change. And is classification based on phenotypes and genotypes, or just phenotypes? Also, there was only one PR strain for classes 3 and 3+ were more identified in these classes? Overall, there was confusion about identification and classification of PRs. (And was PR2 in this paper the same strain as PR2 in Parrino et al, 2017?)

We began this study by isolating a large set of PR mutants. To ensure they were independent, each PR mutant strain was isolated from an independent single colony of the parental *cyr1*∆/∆ strain. We subsequently found that the PR mutants showed phenotypic variation, and could be grouped into 4 classes, based on their phenotypes. We picked 1 or 2 mutants from each class for genome sequencing. Interestingly, all 7 PR mutants contained genetic changes that resulted in loss of expression of one copy of *BCY1*. In addition, each class had a distinct genetic variation, which caused phenotypic differences between classes. We also note that the two different class 1 mutants had different mutations that introduced nonsense codons (S1 Table) confirming that they are independent.

We agree it was striking that the two independent class 2 PR mutants lost the same 276-kb region of chromosome 2. Inspection of the genome sequence revealed a telomere-like sequence (ACATCCGTA) at the 276-kb break point in chromosome 2 (S1 Table). We hypothesize that the presence of this sequence increased the probability of a *de novo* telomere addition, resulting in the 2 independent PR mutants losing the same part of chromosome 2.

Yes, the PR2 mutant was identified and studied in the previous paper. In the present study, we revealed that the PR2 mutant had partial monosomy and trisomy in chromosome 2.

2. In Fig 1 and Fig 2 the authors show nicely that PR strains form filaments in response to several cues. However, the quantification in Fig 2D and E only shows the % filamentous in the presence of GlcNac, not in the control cultures (without GlcNac). It would be more convincing to see the % change in filaments +/- GlcNAc, to know how much more filamentation occurs with GlcNac relative to the control (similar to what the authors showed in the Parrino et al paper, Fig 8B). Some strains appear to show a high % of background filamentation even without GlcNac, which gets to the heart of the question of how much filamentation occurs even without a stimulus in these strains.

We added new plots in S1 Fig to show the % filamentous cells before hyphal induction. Class 2, 3, and 3+ were a little filamentous before adding hyphal inducers, but the percent of hyphal cells increased afterward.

3. In their previous paper on this topic (Parrino et al.), the authors said that no mutations involving BCY1 were identified in faster growing cyr1 pseudorevertants. Were mutations missed? Or were the monosomic regions (changes in copy number) not detected? While not a critical factor, clarification of this would be helpful.

We revised the manuscript to describe this (lines 463-465). The bioinformaticists who carried out the genome sequence analysis for us in the previous study did not detect the changes in ploidy, perhaps because read depth was not as good as in the present study. In the present study we switched to the New York Genome Center and obtained better results. The read depth was over 90X so we could detect loss of one copy of *BCY1* in the PR mutants and could define the breakpoint regions.

4. Could the authors clarify how the bcy1/BCY cyr1/cyr1 strain was made? Was this using the CRISPR system or a different system? In general, more information about strain construction is required. This should include which oligos are used for which constructs / strains, as this is not obvious just from the oligos listed in Table S5. This information could be included in supplementary information if too extensive for the main methods. In addition, how were large CRISPR-mediated deletions confirmed? By PCR of junctions or by whole genome sequencing?

We added the requested information to the Supplemental Methods. We made strain KM11 (*bcy1∆*/*BCY1 cyr1∆*/*∆)* without using the CRISPR system. The parental *cyr1∆/∆* strain was made in the previous study (Parrino et al., 2017) and the NAT selection marker (SAT1-FLP) was removed by marker recycling. The *BCY1* deletion construct was synthesized with the BCY1_NAT_FLP_For and BCY1_NAT_FLP_Rev primers, using the plasmid pGR-NAT plasmid as a template. The construct was transformed into the *cyr1∆/∆* cells and the heterozygous *BCY1* deletion mutants were selected for NAT resistance. Genotype PCR was performed to confirm the large CRISPR-mediated deletion.

5. How does a bcy1/bcy1 cyr1/cyr1 double mutant behave? Has this been tried either by these authors or in other studies (either in Candida or other species)? It might also have been useful to add BCY1 back to the PR mutants to further establish the contribution of gene dosage to growth rates/filamentation, although it is acknowledged that slow growing transformants are likely to have to be identified.

Huang’s group generated *bcy1∆*/*∆* in *C. albicans* (PMID: 28105026). Homozygous deletion of *BCY1* lead to multiple cellular morphologies and promoted the development of elongated and filamentous cells. Interestingly, the *bcy1∆*/*∆* mutant was not stable and could undergo spontaneous switching between the two morphological types, filamentous and smooth colonies.

We made the *bcy1∆*/*∆ cyr1∆*/*∆* mutant and it showed similar phenotype to the Huang’s report. The *BCY1* homozygous deletion improved the growth and filamentation as did the heterozygous deletion did. The mutant was also unstable and produced different colony shapes.

We have shown that the gene dosage effect of *BCY1* improved growth and filamentation in a variety of ways. Genome sequencing analysis identified loss of one copy of *BCY1* allele in all the PR mutants sequenced. RNA-seq showed the haploinsufficiency reduced *BCY1* transcript level by 50% in the PR. Western blot validated the gene dosage effect by showing the 50% reduction of Bcy1 protein level in the PR. Finally, genetic manipulation in chromosome 2 recapitulated the PR phenotypes, confirming that the genetic changes in chromosome 2 improved growth and hyphal induction in the PR mutants.

6. One concern was how the phosphoproteomics data was being used/interpreted. Much of the paper’s more general conclusions rely on the ability to accurately identify all phosphosites and, in several cases, the kinases that produce these phosphosites. I am not convinced that a proteome-wide screen will detect all phosphosites (for technical reasons) and so it still seems possible that PKA activity is the same before/after hyphal induction? Along the same lines, it should be clarified that Yck2 and Hgc1 are predicted to phosphorylate some of the detected sites based on their consensus motifs, and that direct phosphorylation by these factors is not shown in the current study. (And attempts to make phospho-mutations in key residues in Bni1/Mob2 did not show hyphal defects).

Although it is true that proteomics cannot identify all phosphosites when the sample is too complex, thanks to recent technological advance we were able to detect 17,580 phosphopeptides and 2,156 phosphoproteins in the *C. albicans* cells. 268 phosphopeptides were predicted to be PKA substrates based on the consensus motif. We performed GSEA analysis to compare the expression of the 268 phosphopeptides before/after hyphal induction and could not detect a statistically significant difference. This indicates that the PKA activity did not change during hyphal induction. While there could be additional unidentified PKA substrates, we do not think they will change the overall trend of PKA activity estimated from the proteome-wide data. Furthermore, Western blots showed that PKA subunit (Bcy1 and Tpk2) levels remained constant during hyphal induction (S2 Fig). Thus, we did not detect any evidence to support the stimulation of PKA in the PR mutants during hyphal induction.

We modified the text to clarify that Yck2 and Hgc1 were predicted to phosphorylate some of the detected phosphosites based on their consensus motifs (lines 260-262).

7. For Table S3, information lacking as to what is in the table. Terms like “positions in master proteins” are not defined or what the code in this column refers to. Also, what are the units in columns J-Q for abundance?

Table S3 has been revised. “Positions in master proteins” shows UniProt accession number of the original protein and the position of the detected peptide. For example, “A0A1D8PC38 [33-47]” means that this peptide was from the protein A0A1D8PC38 (UniProt accession number) and its location is from 33 to 47 in amino acid. “Modifications in master proteins” shows UniProt accession number of the original protein, and the number and the position of the protein modification (e.g., phosphorylation). The phosphopeptide abundance in columns J-Q is expressed in a label free quantitation (LFQ) unit normalized by protein abundance. We added a “Readme” tab in Data S3 to address the reviewer’s comment.

8. The authors state that the hgc1 null mutant caused a stronger hyphal defect in the PR13 mutant than in the WT control, but this is hard to see from the cell images in Fig S5A. Could a quantitative analysis be included? The same is true for the effect of yck2 deletion on WT/PR13 strain backgrounds. In the case of the yck2 deletion the strains look very pseudophyphal, so are authors distinguishing between hyphae and pseudohyphae, or are both just counted as filaments throughout? This is an important point that should be discussed as it is relevant to all of the filamentation assays.

We quantified the percent filamentous cells in S5B Fig to address the reviewer’s comment. Despite of the abnormal morphology, the *hgc1*Δ/Δ mutant produced a similar number of filamentous cells as the WT control strain (~95%). However, the PR13 *hgc1*Δ/Δ mutant produced a lower level of filamentous cells (36%) compared to the PR13 strain (61%).

We counted both hyphae and pseudohyphae as filaments to make the image analysis consistent. In this criterion, the *yck2*Δ/Δ cells are mostly filamentous in both WT and PR13 backgrounds. However, in the text, we pointed out that the *yck2*Δ/Δ cells displayed an elongated cell morphology that did not switch to a true hyphal morphology in the presence of the hyphal inducers.

9. The text says that translation rates of hyphal TFs increased dramatically during hyphal growth and cite Fig 5E. However, the data in this panel is not significant for hyphal TFs as a group? This should be revised/clarified.

We revised the text to address the reviewer’s comment (lines 284-296). As a group, the difference was not very significant because there was a huge variation in the translation rates of the hyphal regulator TFs. However, translation rates of Ume6, Sfl1, Brg1, Tec1, and Fkh2 increased dramatically during hyphal growth.

10. An overall model showing where things stand in terms of hyphal regulation would help and would be more useful than Fig S6 which did not add much to the paper. This model could clarify what is truly new about the hyphal induction model, given that it seems like pseudorevertants are still dependent on Efg1 for hyphal formation (Parrino et al.) and that Hgc1 and Yck2 were previously shown to regulate filamentation. Is it true that all pseudorevertants still require Efg1 to filament like PR2 shown previously? Overall, the reader is left unclear as to how transcription factors and kinases are envisaged to work together, even if this is still hypothetical.

We thank the reviewer for suggesting a major modification to the summary Fig. 6. We agree that this will strengthen our ability to convey the key conclusions of the manuscript. We now provide a model that integrates the findings of our study and the work of others. A key aspect of this model is that various hyphal inducers and TFs are predicted to induce a new physiological state that promotes polarized filamentous growth, in part due to changes in protein phosphorylation. A short summary of the model is below. It will be described in more detail in the Discussion.

This model indicates that cAMP and adenylyl cyclase are important cellular regulators that can contribute to hyphal growth. But, other factors can also contribute to creating the appropriate physiological state as indicated by the PR mutants.

Similarly, we think that the role of TFs in regulating hyphal growth is to help create the appropriate physiological state. This helps to explain several findings in the literature. One is that although several TFs are clearly important for inducing hyphal growth, they do not appear to regulate the expression of any new proteins directly involved in morphogenesis. Also, many of these TFs can be bypassed under different genetic or environmental conditions. Thus, we propose that the role of these TFs is not to induce a specific gene, but rather they control sets of genes that promote a new physiological state. Consistent with this, the model highlights new data from our manuscript showing that global transcriptional regulators (mediator and transcription elongation factor complex) contribute to hyphal regulation. Also, it indicates how changes in the level of different TFs occurs in response to hyphal inducers can help to create the appropriate cellular state.

The model also shows how changes in phosphorylation on consensus sites for Hgc1-Cdc28 and Casein kinase 1 are linked with hyphal induction. We propose that these kinases are activated in part by changes in the cell physiology that are induced. Although previous studies have implicated these protein kinases, we note that our study is the first to show that they can be activated in a *cyr1*Δ mutant.

This study also challenges the old model that cAMP-PKA signaling activates transcription of hyphal genes by phosphorylating the hyphal regulator TFs such as Efg1. The PR mutants could form hyphae without cAMP signaling and the PKA subunits were constantly expressed. In addition, PKA activity was estimated to be similar before/after hyphal induction. Our phosphoproteomic data did not detect the previously reported Efg1 phosphosites and it is in consistent with the reports of Willger et al. and Cao et al. Thus, we found no evidence to support the stimulation of Efg1 through the cAMP-PKA pathway. As a new model, we here suggest that basal level of PKA activity and hyphal regulator TFs create a new physiological state for hyphal induction. We have not tested the role of EFG1 in the other PR mutants. As described above, since deleting EFG1 causes changes in the expression of a large number of genes we think that this shifts the cells into a different physiological state that does not promote filamentous growth.

**Part III – Minor Issues: Editorial and Data Presentation Modifications**
Please use this section for editorial suggestions as well as relatively minor modifications of existing data that would enhance clarity.

**Reviewer #1:**

1. Most deletions by the CRISPR-Cas9 system are heterozygous. Can the authors confirm that all the chromosome deletions shown in Figure 2 are actually heterozygous mutations?

We added more information to the Methods to describe how we confirmed that the deletion mutants in Fig 2 are heterozygous (lines 431-436). As described in a previous paper (Min, 2016), two PCR detection strategies were designed to detect the wild-type allele and the deletion allele. More than 10 independent transformants were tested for each deletion and around half of them had both WT and deletion alleles (heterozygous deletion). Interestingly, we could not find any homozygous deletion mutant although we used CRISPR to increase the transformation frequency. We think this is an expected result as the large homozygous deletions in chromosome 2 would have a detrimental effect on the cells because there are essential genes including the global transcriptional regulators we studied.

2. Lines 212-213. There is a discrepancy about Efg1 phosphorylation by the PKA pathway. Aside from pointing out this discrepancy it would be useful to have some explanation.

We added more explanation to address the reviewer’s comment. In our opinion, this difference seems to arise from the different experimental techniques used. Bockmuhl et al. (PMID 11290709) and Wang et al. (PMID 19528234) reported that T206 and T179 positions are phosphorylated by PKA and Cdc28 kinase, respectively. However, these results were based on genetic studies (such as phosphomutants) and they did not use mass spectrometry to confirm the phosphorylation. Recently, Willger et al. (PMID: 25750214) and Cao et al. analyzed the phosphoproteome in *C. albicans* hyphae (PMID: 28370450). Surprisingly, they did not detect the previously reported phosphosites of Efg1. In this study, we also did not detect phosphorylation of either T179 or T206. Interestingly, Efg1 was dephosphorylated during hyphal induction at a different set of sites in the C-terminal prion-like domain (PrLD), which supports the assembly of TF complexes by formation of phase-separated condensates (Frazer et al. PMID 32719507). In summary, three independent studies have not detected phosphorylation of Efg1 which was implicated from genetic evidence. This indicates that Efg1 is not phosphorylated at the previously reported sites in response to hyphal induction (lines 226-236).

3. S3 Figure: why is the spt5 mutant reduced for filamentation but the srb9 spt5 mutant shows enhanced filamentation? Some additional commentary and explanation here would help.

We added additional explanation in the text (lines 182-186). We think that this is due to genetic interactions. One way to explain this is to use the multiplicative model that was used by the Krysan lab when they studied the interaction of *C. albicans* biofilm transcription factors by generating double heterozygous mutants of the network genes (PMID: 28793308).

Since the *spt5*Δ mutant lead to a reduction in filamentation by 0.8 and the *srb9*Δ mutant lead to an increase in filamentation by 1.3, the multiplicative model predicts that the *srb9*Δ *spt5*Δ double heterozygous mutant would have a phenotype of 0.8 × 1.3 = 1.04. However, the *srb9*Δ *spt5*Δ mutant showed an increase in filamentation by 1.4. According to the multiplicative model, double mutants with less severe phenotypes than the expected phenotype suggest that the two genes function in the same pathway (diminishing returns effect) or compensate for one another (PMID: 18305163). This result is in consistent with the fact that *srb9*Δ and *spt5*Δ function in the general transcription machinery.

4. S5 Figure: the phospho-mutants of Mob2 and Bni1 fail to show a filamentation defect. It would help to have some additional evidence that non-cAMP-dependent phosphorylation of target proteins is important for driving filamentous growth.

It may be that the phosphorylation of a larger set of proteins acts in concert to promote filamentous growth such that blocking the phosphorylation of one protein does not have a strong effect. However, we note that we provide evidence for the role of Hgc1-Cdc28 and casein kinase 1 (Yck2) in hyphal growth. In addition, previous studies have identified proteins whose phosphorylation by cyclin-dependent kinases is important for hyphal growth (see references 26-34).

5. S5 Figure: the yck2 mutant in the WT background generates filaments but in the PR13 background this is not the case. Why? This should be discussed.

The photos in the figure show only a small set of cells. Therefore, we performed quantitation for the hyphal induction assay and added the data in S5B Fig. The *yck2*Δ/Δ mutant was filamentous in the PR13 background and the % filamentous cells was not significantly different from the WT background.

6. Figure S2A: Why was total protein used for normalization rather than a standard loading control (eg: Act1)? How could quantitation be performed if this not a single band on the gel?

In our recent experience we have found that, although actin can be a good control for cells grown under similar conditions, it is not always a good control for when they are stimulated by a change in conditions. (PMID: 33468576).

We quantified Coomassie stained gels with a using a digital Odyssey scanner and ImageStudio software (LI-COR Biosciences). We quantified based on the bands in the whole lane for total protein, rather than just one protein that could be affected by hyphal induction conditions.

**Reviewer #2:**

Figure 2C. The wide variation in doubling times of the cyr1 mutant in six independent experiments is puzzling. Were different strains or colonies used? Or there are other explanations.

Yes, the *cyr1*Δ/Δ showed wider variation in doubling times than the other strains. We used six different colonies of the *cyr1*Δ/Δ strain, and the assays were done on different days. We speculate that this is variation could be due to the genomic instability of the *cyr1*Δ/Δ mutant. Even in a short time frame (< 24 h), it could start to undergo spontaneous mutation and chromosomal loss to improve the fitness. Thus, it was possible that some of the *cyr1*Δ/Δ cultures could have contained spontaneous PR mutants during the doubling time measurement.

Lines 66-67 and 276-278. The authors argue that because the cyr1 deletion mutant grows very slowly, it does not support the essential role of Cyr1 in hyphal growth. The statements are not entirely correct. There are point mutations in the LRR domain of Cyr1 that allow normal yeast growth but not hyphal growth. Xu et al., Cell Host & Microbe 4:28-39; Wang and Xu, Commun Integr Biol 1, 137-139.
Line 76-77. The statement ‘transcriptional analysis of hyphal induced genes has not revealed the mechanism of hyphal induction’ is not fair. While it is true that the vast majority of HSGs are not required for hyphal growth, some of them play critical roles in hyphal development, such as UME6 and HGC1. This study of the PR mutants also confirms the essential role of HGC1 for hyphal development.

Lines 66-67 state that the phenotype of *cyr1*Δ mutants grow slowly, as is shown in Figure 2 and in published data from several other labs. It seems that the reviewer’s point is that there are special alleles of CYR1 that grow well but do not form hyphae. We therefore revised this section to better explain our point that although cAMP is sufficient to induce hyphae, other pathways can also contribute. Now we discuss this in the first paragraph of the Discussion section.

We have revised lines 77-81 to make the manuscript clearer that hyphal-specific genes are not directly involved in morphogenesis. *HGC1* encodes a cyclin and *UME6* encodes a transcription factor. The point we are trying to make is that they do not participate directly in morphogenesis.

Line 86. It is not appropriate to use these studies to argue against the role of cAMP in regulating hyphal growth. Cdc28 is far downstream along the hyphal induction pathway; and the Cdc28/Hgc1 is activated by cAMP signaling. Please modify this statement.

We modified this statement to say that there are other protein kinases that play a role in promoting hyphal morphogenesis that are not directly regulated by cAMP.

Line 179. The authors should be careful when they say ‘cAMP signaling is not necessary for hyphal growth or transcription regulation of genes during hyphal induction’. cAMP signaling includes necessarily the targets of cAMP such as PKA. In the PR mutants, PKA is upregulated due to the partial loss of Bcy1, which could, in a way, support the importance of cAMP signalling (even only to ensure a basal level of activity). In a signal pathway, activation of a downstream component often renders upstream elements unnecessary, but it does not mean the pathway is not needed. Here, the authors merely demonstrate that the PR mutants retain the ability to activate hyphal-specific genes in response to their hyphal induction condition.

We modified the sentence by replacing cAMP signaling with adenylyl cyclase. (We had been thinking of a narrower interpretation of cAMP signaling to mean just the activation of cAMP by adenylyl cyclase.)

Lines 186, 190. The transcriptome profile similarities are relative. By eyeing the heatmaps, one sees more differences than similarities even between the most similar ones. The analyses merely show that some are more similar than others. The description ‘similar patterns’ or “highly similar’ is arbitrary and should be rephrased.

The manuscript was modified to better indicate that clustering analysis was used to determine the similarity in the transcriptomes (Figure 3B). All uninduced WT and PR strains clustered together. Similarly, all induced WT and PR strains clustered together. In contrast, the *cyr1*Δ mutant did not cluster with the other strains either induced or uninduced. We modified lines 204-209 to indicate that PR13 was more similar to the WT because it had a lower basal level of hyphal gene expression than did PR12 (as can be seen in Fig. 3C).

Lines 303-305. The constitutive growth of the *TUP1* mutant does not argue against the role of TFs in hyphal growth. In the absence of the general repressor Tup1, some of the hyphal-promoting TFs are upregulated.

The Discussion section was revised extensively. However, we note that we have deleted the statement about TUP1 and replaced it with a reference to a study by Wartenberg et al. showing that a hyphal growth was restored in a *cph1∆/∆ efg1∆/∆* mutant by a mutation in the SSN3 subunit of the Mediator complex (Wartenberg 2014, PMID: 25474009).

Figure 3c legend. The ‘Grey boxes’ are not obvious. I only see different shades of blue and yellow. It is better to state that values above or below a specific number are insignificant. The results (the CYR1 row) show that CYR1 mRNA was detected in the CYR1-deletion and PR mutants, albeit at very low levels?

We modified the Fig 3C and the legend to make it more visible. Now grey numbers indicate if there are no significant differences.

We think the very low level of *CYR1* mRNA detected in strains lacking *CYR1* is due to the index switching (“Index hopping") of multiplexed samples on the Illumina platforms. Multiplexing is efficient for analysis of multiple samples in the same manner; however, with multiplexing, the potential for index hopping is present regardless of the library prep method or sequencing system used. Index hopping may result in assignment of sequencing reads to the wrong index during demultiplexing, leading to misalignment. According to Illumina, typical levels of index hopping range from 0.1-0.2% so this explains why the *cyr1*Δ/Δ and PR mutants had low background signal (< 0.1% of the WT level) of *CYR1* reads. Illumina has released a website that address this issue. https://www.illumina.com/techniques/sequencing/ngs-library-prep/multiplexing/index-hopping.html

Figure 4c is incomplete. Readers want to see the comparison between WT+GlcNac vs. WT-control and PR13+GlcNac vs. PR13-control for substrates of both Cdc28 and Yck2 kinases.
Was only the WT strain used in the experiments described in Figure 5? Are there any evidence that the protein level of these TFs are increased in the PR mutants?

We modified Fig 4C and 4D to address the reviewer’s helpful comments. Now, Fig 4C shows the comparison between samples for substrates of both Cdc28 and Yck2 kinases. We added one more row for substrates of Cdc28 & Yck2 in Fig 4D. The new data demonstrate that the substrates of Cdc28 & Yck2 were phosphorylated during the hyphal induction in the WT and PR13 cells. This indicates Cdc28 & Yck2 can function in a cAMP-independent mechanism.

Fig 5 shows translation rates in the WT strain. We added the PR13 data in S6 Fig. In the PR13 mutant, there was an increase in translation rates of *RIM101*, *RFG1*, and *BRG1*. We believe the PR13 mutant showed the different pattern because it was weakly filamentous even before hyphal induction. As a result, there was a higher basal level of some TFs and consequently less of an apparent increase after stimulating with GlcNAc (S1 Fig).

**Reviewer #3:**

1. I suggest the authors could consider renaming the strains so that they are more informative as random numbers (PR13, PR12, PR18 are not meaningful). The new names could match the class from which they are from 1-1, 2-2 etc….

We tried some different strategies and decided to keep the PR designation as it is, but we added the class designation in the figures to make them more recognizable for readers. (One reason is that we wanted to avoid confusion with the previously published PR2 strain, which is a class 3+ strain.)

2. Lines 212-213. Are the authors saying “inconsistent” or “consistent”?

Sentence was revised. (Unfortunately, there was a missing word that caused confusion.)

3. Wrong figure is cited on line 169 - should be Fig S2.

We think the citation is correct. S3 Fig shows gene mapping analysis of the 10-kb region.

4. The gene name for Yck2 on line 232 is confusing as says “yeast casein kinase 1 (Yck2)”. This could be clarified by saying casein kinase I family?

We revised the sentence as suggested.

5. In many figures the statistical significance is shown relative to the WT or parental strain, but it is also relevant to see significance relative to other strains in a series. For example, in Figure 2 it would be interesting to see the pseudorevertants compared to the cyr1 mutant as well as the WT. This was also true of other panels in the paper.

We added the comparisons between *cyr1*Δ/Δ and the other strains in the figure legend. The doubling time of the PR mutants was significantly shorter when compared to the *cyr1*∆/∆ background (p < 0.01). (We also added this information to the legend for Fig. 1)

6. In the Author Summary and Discussion, it is stated that translational regulation of key transcription factors is a novel mechanism of hyphal regulation. However, translational regulation of several of the key TFs has previously been shown so that is not a truly novel finding. A revision of the text would help here.

It seems that the reviewer is referring to the studies on the 5’ UTR regions of *EFG1* and *UME6* (Desai et al. PMID: 29976646, Childers et al. PMID: 24601998). Yes, the long 5’ UTR region promoted translation of Efg1 while it inhibited the translation of Ume6. These results indicate that the 5’ UTR can play important but opposing roles in translation efficiency. However, in our present study, we found no correlation between 5’ UTR length and translation rate in the hyphal regulator TFs. For example, *BRG1* does not have long 5’ UTR regions (~100 bp) but it was translationally regulated, implicating a novel mechanism. We believe that differential translation of transcription factors could be an important mechanism that contributes to hyphal growth and the findings presented here open up exciting opportunities for future investigations.

7. On 237, the text reads that “… both kinases [Cdc28 and Yck2] phosphorylated 11 sites in 8 genes….. Kinases phosphorylate proteins.

We fixed the mistake.

8. Could the authors clarify why the strains were first grown in SC+ Galactose media for transcriptomic analysis and also for proteomics analysis?

We used galactose for the carbon source because glucose represses the expression of the GlcNAc transporter, but galactose does not PMID: 17192409. The use of galactose therefore permits a strategy where cells can be grown to an appropriate density and then GlcNAc added to induce hyphal morphogenesis. This avoids triggering gene expression of changes in protein levels that could be caused by having to wash out the glucose medium prior to induction. We think that this helped to account for the reproducibility of our RNA-seq and proteomic results. We added a description to the Methods (lines 388-390) to explain the reason why galactose was used.

9. In discussing the SRB9 and SPT5 data it would be relevant to include a mention that SSN3 mutants also impact filamentation phenotypes, as there seems to be a pattern emerging with mutation of transcriptional co-factors impacting filamentation (Wartenburg et al., 2014).

We thank the reviewer for this helpful suggestion and have cited it in several places in the manuscript. We came across the paper right after the submission and have been regretting that we missed the opportunity to cite it. It is very interesting that the two independent studies found mutations in the subunits of general transcription machinery.
