# Supplementary material for: Integrative multi-omics profiling reveals cAMP-independent mechanisms regulating hyphal morphogenesis in Candida albicans
Source: PLoS Pathog. 2021 Aug 16;17(8):e1009861. doi: 10.1371/journal.ppat.1009861 (PMC8389844; doi:10.1371/journal.ppat.1009861)
Supplement: Attachment — Submitted filename: Response to Reviewers_July22.docx [file ppat.1009861.s018.docx]

**Response to Reviewers’ Comments:**

We thank the reviewers for their careful reading of our manuscript and helpful suggestions. We have addressed the comments as follows:

**Part I**

Reviewer #1: In general, the authors have done a good job in addressing my concerns and the revised manuscript is significantly strengthened. However, the authors have still not provided independent validation for the multi-omics data as requested. While it is reassuring to know that these data are highly consistent, the exact number of replicates was not clearly specified. From the legend for Figure 4B it appears that only 2 replicates were used for the proteomics and phosphoproteomics experiments.

Yes, there were 2 biological replicates in the omics experiments. The number of replicates is now stated in the legends to Fig. 3 and Fig. 4.

Reviewer #3: The revised version of the Min et al. manuscript addresses several of the issues raised by reviewers in the original submission, and the edited version is much improved. There were still some points that could use further clarification as outlined below. Most of these request editing of the text or data analysis rather than additional experiments.

**Part II – Major Issues: Key Experiments Required for Acceptance**

Reviewer #1: (No Response)

Reviewer #3: 1. The authors emphasize that the number of phosphopeptides assigned to PKA activity do not change between yeast and hyphae. But are the exact same phosphopeptides present in both conditions? It appears that GSEA analysis shows that the functional class of proteins phosphorylated do not change, but is it possible a change in the PKA pattern could be important even if the total number of PKA phosphopeptides do not? This was hard to tell by looking at the tables.

For GSEA analysis, we compared the level of the phosphorylation on peptides between basal and induced conditions, not the number of different phosphopeptides. The phosphopeptides with PKA substrates motifs were not significantly up or down-regulated during hyphal induction. This result supports the conclusion that PKA kinase activity was constant.

2. The recent Mundodi et al paper (PMC7849906) that examines translation efficiencies associated with filamentation should be mentioned/discussed. Some comparison of the current data set with the Mundodi one would be interesting as to whether the same genes/trends are identified. A cursory comparison appears to show different trends, albeit using different experimental approaches. While the current paper shows that the protein-to-mRNA ratio of UME6, SFL1, BRG1, TEC1, and FKH2 increased dramatically during hyphal induction (while mRNA levels did not), Mundodi shows that the TE (translational efficiency) for several of these genes decreased during hyphal induction (e.g. TEs for UME6, SFL1, BRG1, FKH2) although the decrease was not significant. Can the authors explain why TE is reduced or unchanged, whereas protein/mRNA ratios increase for these genes? Is this due to induction of hyphae using different conditions? This makes it hard to know whether translation of these regulatory genes is really up or down under hyphal conditions.

We cited the Mundodi et al. paper in the Results section. Please see lines 303-312.

There were many differences between our study and the paper by Mondodi et al. However, we think that a major factor is likely the differences in experimental methods used to measure the protein synthesis rate. Mundodi et al. used ribosome profiling, which measures ribosome density on mRNA transcripts. For these studies, Ribo-seq reads are divided by amount of total transcript to yield a factor termed the translational efficiency (TE) of the transcript. Assuming that the average translation elongation rate is similar for different genes, ribosome profiling provides an indirect readout for protein synthesis. However, due to biological factors such as different translation elongation speeds, the amino acid composition of the nascent peptide chain and ribosome queuing, as well as data normalization issues, ribosome densities (TEs) as measured by ribosome profiling do not necessarily reflect the protein output of the ribosome. In contrast, we measured the protein-mRNA ratio, which is a more direct readout for protein synthesis rate. The different results suggest there could be changes in the translational mechanisms during hyphal growth.

3. In terms of the overall model, could the authors clarify what is meant by a new “physiological state” that supports hyphal formation? Presumably transcription of certain targets is necessary to prime the cell, but this priming is seen as necessary but not sufficient for hyphal induction in this model? Otherwise this term seems a but mysterious.

At present, we cannot describe specific factors that are critical to the altered physiological state of the cell. However, we addressed this starting in paragraph 2 of the Discussion, in which we propose that “these data suggest a new model that TFs are important for creating the proper physiological environment for cells to undergo hyphal growth, rather than inducing specific target genes that are directly involved in promoting filamentous morphogenesis”. The next paragraph notes that although altered expression of some TFs can induce hyphae, these changes in transcription are associated with broad changes in cell physiology, which are expected to alter the state of the cell. The subsequent paragraph further develops this idea by proposing that this altered physiological state activates protein kinases such as Cdc28 and Yck2 that promote hyphal morphogenesis.

Part III – Minor Issues: Editorial and Data Presentation Modifications

Please use this section for editorial suggestions as well as relatively minor modifications of existing data that would enhance clarity.

Reviewer #1: (No Response)

Reviewer #3: One remaining question still concerns how cyr1 revertants were divided into the 4 PR classes. It is still unclear if these are being classified based off growth rates and filamentation percentages alone, or if other phenotypes are being use for classification (or if genotypes are also used). This needs to be described clearly for the reader. It is also still unclear if multiple PR revertants were identified for each class of the classes or if some classes are really singletons.

Yes, the PR mutants were separated into four classes based on their growth rates and hyphal phenotypes alone. This is stated in the first paragraph of the Results section (lines 107-121) and the data are presented in Table 1. Below, we inserted a simplified version of Table 1, which we hope will make it easier to see that the different classes behave differently. We subsequently picked representative isolates from each class for genome sequencing. The genome sequencing results confirmed that the different classes had distinct chromosomal changes, but this was not used to place the PR mutants into different classes.

| Strain | PR class | Growth rate | Filamentation |
| --- | --- | --- | --- |
| WT |  | ++++ | ++++ |
| *cyr1∆*/*∆* |  | + | - |
| PR13, PR19 | Class 1 | ++++ | ++ |
| PR12, PR14 | Class 2 | +++ | ++++ |
| PR18 | Class 3 | ++ | ++++ |
| PR16, PR2 | Class 3+ | ++ | +++ |

The authors state that on lines 50-52 that “post-transcriptional mechanisms regulate the levels of a set of key transcription factors that are important for hyphal induction, suggesting a novel form of translational regulation.” I am still not sure that this can be considered “novel” given that post-transcriptional mechanisms have already been linked to some of these genes (via their UTRs) and that the mechanism involved for other transcription factors has not been identified. I would simply suggest that removing the term “novel” would be safer to avoid the risk of seeming to over sell the conclusions.

We removed the term “novel” to address the reviewer’s comment. Please see lines 52 and 759. (However, we think that it is likely that there is a distinct form of translation regulation, since our analysis of the data indicates that it is not dependent on a long 5’ UTR.)

The authors provide data showing that some of the PR strains are highly filamentous even without an inducing cue and it seems more could be made of this point. It appears important that basal rates of filamentation are now very high in some PR classes such as in class 2/PR12.The clarity of the phosphorylation proteomics data is improved over the initial submission (Data S3). However, it was not clear to me why not all rows have a peptide (15mer) listed in column X? Or why some rows have multiple 15 mers listed? Also in Data S3 it shows that some peptides are phosphorylated at multiple positions – sometimes these positions are indicated in column D and sometimes they are not?

We revised the text to describe the basal and induced filamentation rates for the gene mapping experiments. In our new summary of the results, we address the effects of deleting different regions of chromosome 2 on both the basal and GlcNAc-induced levels of filamentous growth. Please see lines 176-181.

Regarding the phosphoproteomics questions, we revised the table to correct a formatting issue for the display of the peptides. In addition, we note that most of the phosphopeptides have one phosphorylation site, so they are represented in the table as a single 15-mer sequence. However, some phosphopeptides have more than one phosphorylation site identified, so they have multiple 15-mer sequences. In other cases, although a phosphorylation site was detected, it might not be possible to identify the precise site of modification (PMID: 23447708). For example, same-sequence peptides phosphorylated on different residues have identical intact masses. When fragmented for MS/MS, only fragments resulting from breakage points located between the two sites can distinguish them. All other fragment ions are the same for these two distinct phosphopeptides. If MS/MS cannot detect the key fragments, the precise site of phosphorylation cannot be identified. Therefore, the 15-mer sequence could not be shown for these phosphopeptides although we know they had phosphosite(s).

Data table S4 needs a similar ReadMe introduction to Data table S3 to provide clarity as to what is being shown.

We added the ReadMe tab in Data table S4.

It would be useful if the methods included the volumes of cultures/cells used for transcriptomic and proteomic analyses, as well as for western blotting analyses.

We added more information in the Methods section. Please see lines 409, 486, 513-514, and 544.

Regarding the PR2 mutant that was previously described as not have any genetic changes linked to phenotypic differences, it does appear the authors hide this fact by only adding a sentence to the Methods (463-465). This really should be mentioned more plainly in the Results and not in the Methods where most readers would not find this fact. I do not see this as a problem that needs to be hidden.

The manuscript was revised to describe that the changes in Chromosome 2 ploidy for the PR2 strain were newly detected in this study and not reported in our previous manuscript. Please see lines 131-134.
